# The scaffold protein WRAP53β orchestrates the ubiquitin response critical for DNA double-strand break repair

**DOI:** 10.1101/gad.246546.114

**Published:** 2014-12-15

**Authors:** Sofia Henriksson, Hanif Rassoolzadeh, Elisabeth Hedström, Christos Coucoravas, Alexander Julner, Michael Goldstein, Gabriela Imreh, Boris Zhivotovsky, Michael B. Kastan, Thomas Helleday, Marianne Farnebo

**Affiliations:** 1 Department of Oncology-Pathology, Cancer Centrum Karolinska (CCK), Karolinska Institutet, Stockholm 171 76, Sweden;; 2 Department of Pharmacology and Cancer Biology, Duke University School of Medicine, Durham, North Carolina 27710, USA;; 3 Institute for Environmental Medicine, Karolinska Institutet, Stockholm 171 77, Sweden;; 4 Science for Life Laboratory, Division of Translational Medicine and Chemical Biology, Department of Medical Biochemistry and Biophysics, Karolinska Institutet, Stockholm 171 65, Sweden

**Keywords:** WRAP53β, WD40 scaffold, DNA repair, ubiquitin, RNF8 E3 ligase, MDC1

## Abstract

The WD40 domain-containing protein WRAP53β (WD40 encoding RNA antisense to p53; also referred to as WDR79/TCAB1) controls trafficking of splicing factors and the telomerase enzyme to Cajal bodies, and its functional loss has been linked to carcinogenesis, premature aging, and neurodegeneration. Here, we identify WRAP53β as an essential regulator of DNA double-strand break (DSB) repair. WRAP53β rapidly localizes to DSBs in an ATM-, H2AX-, and MDC1-dependent manner. We show that WRAP53β targets the E3 ligase RNF8 to DNA lesions by facilitating the interaction between RNF8 and its upstream partner, MDC1, in response to DNA damage. Simultaneous binding of MDC1 and RNF8 to the highly conserved WD40 scaffold domain of WRAP53β facilitates their interaction and accumulation of RNF8 at DSBs. In this manner, WRAP53β controls proper ubiquitylation at DNA damage sites and the downstream assembly of 53BP1, BRCA1, and RAD51. Furthermore, we reveal that knockdown of WRAP53β impairs DSB repair by both homologous recombination (HR) and nonhomologous end-joining (NHEJ), causes accumulation of spontaneous DNA breaks, and delays recovery from radiation-induced cell cycle arrest. Our findings establish WRAP53β as a novel regulator of DSB repair by providing a scaffold for DNA repair factors.

## Abstract (precis)

The WD40 domain-containing protein WRAP53β controls trafficking of splicing factors and telomerase to Cajal bodies. Here, Henriksson et al. demonstrate that WRAP53β rapidly localizes to double-strand breaks (DSBs) in an ATM-, H2AX-, and MDC1-dependent manner. WRAP53β targets the E3 ligase RNF8 to DNA lesions by facilitating the interaction between RNF8 and its upstream partner, MDC1, in response to DNA damage. Loss of WRAP53β impairs DSB repair by both homologous recombination and nonhomologous end-joining, causes accumulation of spontaneous DNA breaks, and delays recovery from radiation-induced cell cycle arrest.

Proper repair of DNA double-strand breaks (DSBs) is critical for the maintenance of genome stability and prevention of disease; e.g., neurodegeneration, premature aging, and cancer (Jackson and Bartek 2009). The DNA damage response (DDR) involves a coordinated series of events that results in the assembly of DNA repair proteins at sites of DNA damage. One of the earliest events in this signaling cascade is the phosphorylation of the histone variant H2AX at Ser139 (referred to as γH2AX) by the ATM/ATR/DNA-PK kinases (Falck et al. 2005). Upon binding to γH2AX, the MDC1 protein mediates signal expansion on the DNA molecule (Stucki et al. 2005) and serves as an anchor for subsequent recruitment of the E3 ubiquitin ligases RNF8 and RNF168. RNF8 is the first E3 ligase to be recruited to DSBs, which, in conjunction with RNF168, ubiquitylates flanking chromatin (including histones H2AX and H2A) and promotes efficient assembly of the downstream repair factors 53BP1, BRCA1, and RAD51 at the site of damage (Huen et al. 2007; Mailand et al. 2007; Marteijn et al. 2009; Meerang et al. 2011). The DDR involves accumulation of DNA repair factors at DNA lesions, a process mediated largely by protein interactions.

Scaffold proteins, among which some of the most important are the WD40 domain proteins, tether molecules together and provide backbones for the assembly of signaling complexes. Through their unique WD40 repeats (each repeat consisting of ∼40 amino acid residues, including a tryptophan W and aspartic acid D C-terminal dipeptide), these proteins can interact with several partners simultaneously in a nonexclusive manner, thereby facilitating protein interactions (Stirnimann et al. 2010; Xu and Min 2011). One such example is PALB2, which provides a scaffold for interactions between BRCA1, BRCA2, and RAD51, thereby anchoring BRCA2 to sites of DNA damage. Defects in PALB2 lead to impaired formation of this complex and defective homologous recombination (HR) repair, highlighting the critical role played by scaffold proteins in the DDR (Xia et al. 2006; Lan et al. 2009; Sy et al. 2009).

WRAP53β (WD40 encoding RNA antisense to p53; also referred to as WDR79/TCAB1), another WD40 domain protein, facilitates interactions between factors involved in splicing and telomere elongation and their localization to nuclear organelles known as Cajal bodies. Accordingly, loss of WRAP53β leads to collapse of these structures and mislocalization of associated factors (Tycowski et al. 2009; Venteicher et al. 2009; Mahmoudi et al. 2010). In addition to the WRAP53β protein, the *WRAP53* gene encodes a regulatory RNA (WRAP53α) that is produced by usage of an alternative start point for transcription. Although this RNA controls the response of p53 to cellular stress, WRAP53β acts independently of WRAP53α and does not play a role in the regulation of p53 (Farnebo 2009; Mahmoudi et al. 2009).

Aberrations in WRAP53β have been linked to several genetic disorders. For example, inherited mutations in WRAP53β that affect its WD40 domain cause dyskeratosis congenita, a disorder involving bone marrow failure, premature aging, and cancer predisposition (Zhong et al. 2011). Moreover, SNPs in *WRAP53* or altered expression of the protein itself are associated with elevated risk for a variety of sporadic tumors and radioresistant head and neck cancer cells, hematoxicity, and disturbed DNA repair in workers exposed to benzene (Garcia-Closas et al. 2007; Lan et al. 2009; Schildkraut et al. 2009; Mahmoudi et al. 2011; Medrek et al. 2013; Garvin et al. 2014). Furthermore, patients with spinal muscular atrophy, a neurodegenerative disorder that is the leading genetic cause of infant mortality worldwide, exhibit loss of WRAP53β function (Mahmoudi et al. 2010).

Intriguingly, neurodegeneration, aging, and cancer are all processes linked to accumulation of DNA damage. Although this suggests a role for WRAP53β in DNA repair, this role remains unknown. It is noteworthy in this context that WRAP53β has been identified in several proteomic and genome-wide siRNA screens designed to detect factors associated with DDR (Matsuoka et al. 2007; Paulsen et al. 2009; Adamson et al. 2012). These links, together with WRAP53β’s function as a scaffold protein, prompted us to ask whether WRAP53β is involved in the assembly of repair factors at sites of DNA damage and whether loss of this function impairs DNA DSB repair.

## Results

### WRAP53β is recruited to sites of DNA damage in an ATM-, H2AX-, and MDC1-dependent manner

**Figure 1.**
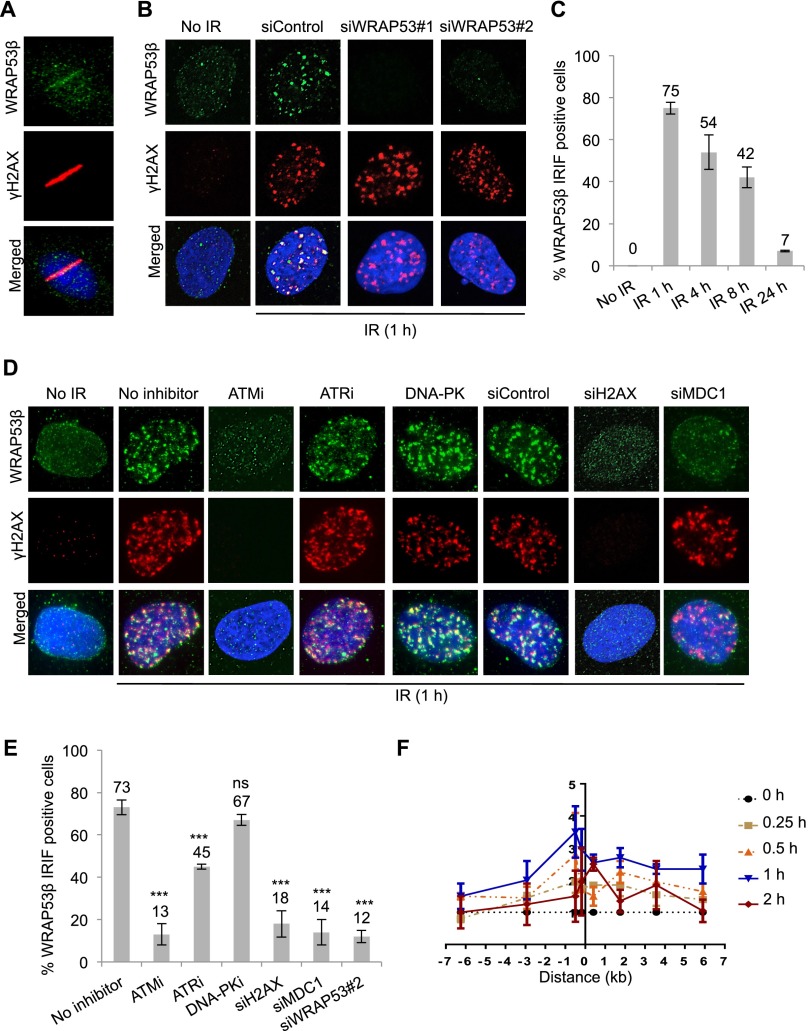
WRAP53β accumulates at sites of DNA damage in an ATM/H2AX/MDC1-dependent manner. (*A*) U2OS cells were microirradiated, fixed 5 min later, and immunostained for WRAP53β and γH2AX, a marker for DNA DSBs. Nuclei were stained with DAPI in all immunofluorescence experiments. (*B*) U2OS cells were treated with siControl or two different WRAP53β targeting oligonucleotides (siWRAP53#1 and siWRAP53#2) for 48 h, irradiated (6 Gy, 1-h recovery) or left untreated, fixed after pre-extraction with cytoskeleton (CSK) buffer, and immunostained for WRAP53β and γH2AX. (*C*) U2OS cells were irradiated (6 Gy), fixed, and immunostained for WRAP53β at the indicated time points. Quantification is given as the percentage of the 100 cells counted in each experiment whose nuclei contained WRAP53β IRIF. (*D*) U2OS cells were treated with the inhibitors or siRNAs, as indicated, for 6 h or 48 h, respectively; irradiated (6 Gy, 1-h recovery); fixed after pre-extraction with CSK buffer; and immunostained for WRAP53β and γH2AX. (*E*) Quantification of the results in *D*, as the percentage of the 100 cells counted in each experiment whose nuclei contained WRAP53β IRIF. The error bars depict the SEM; *n* = 3; (***) *P* < 0.001 as determined by Student’s *t*-test. (*F*) ChIP assay showing the recruitment of WRAP53β to the I-PpoI-induced DSB at chromosome 1 in MCF7 cells stably expressing ddI-PpoI. The time indicated is hours after the addition of 4-OHT. The I-PpoI cleavage site on chromosome 1 is located at distance 0. Cells were cultivated in medium containing 0.1% FBS for 24 h before DSB induction. Data are shown as the mean of two independent experiments. The *Y*-axis displays the fold change in relative occupancy normalized to the control.

To elucidate the involvement of WRAP53β in the DDR, we initially laser-microirradiated U2OS cells and observed a rapid relocalization of WRAP53β to DNA lesions. WRAP53β was present at DNA lesions within a few minutes (Fig. 1A), placing this protein high upstream in the DNA damage signaling cascade. This localization of WRAP53β at DNA damage sites was observed in other cell types, including human fibroblasts and H1299 lung cancer cells, and with five different antibodies against WRAP53β (Supplemental Fig. 1A,B). One of the WRAP53β antibodies, mouse monoclonal α-WDR79 clone 1F12, revealed formation of WRAP53β foci in response to ionizing radiation (IR) as well as enrichment of WRAP53β in Cajal bodies, confirming its reliability (Supplemental Fig. 1C). Furthermore, the WRAP53β foci clearly overlapped with γH2AX, and the staining was specific, since it could be eliminated by siRNA oligos targeting WRAP53β (Fig. 1B). These WRAP53β foci appeared rapidly following exposure to IR and were dissolved gradually over a period of 24 h, a time course similar to that of γH2AX foci (Fig. 1C).

We next set out to determine the mechanism for WRAP53β accumulation at DNA lesions and tested for the contribution of three major kinases involved the early steps of the DDR: ATM, ATR, and DNA-PK (Stiff et al. 2004). This revealed that inhibition of ATM activity impaired the recruitment of WRAP53β to DNA damage sites, whereas DNA-PK and ATR were not strictly required for this localization (Fig. 1D,E). Furthermore, accumulation of WRAP53β at DSBs was abrogated by siRNA knockdown of H2AX and the downstream factor MDC1, both of which serve as anchors for recruitment of downstream factors to DSBs (Fig. 1D,E).

We also investigated the spatiotemporal dynamics of WRAP53β recruitment to the DSB by introduction of DSBs at defined sites in the genome using the I-PpoI homing endonuclease (Goldstein et al. 2013). Chromatin immunoprecipitation (ChIP) revealed that WRAP53β is recruited directly to the break site and, to a lesser extent, to the regions surrounding the DSB (Fig. 1F; Supplemental Fig. 1D). Accumulation of WRAP53β peaked 1 h after ddI-PpoI induction, and WRAP53β disappeared in the DSB surrounding regions 1 h later, a time course that mimics the kinetics of DNA breakage and repair observed in this model system (Goldstein et al. 2013). Interestingly, WRAP53β remained at the break site beyond the 2-h time point after ddI-PpoI induction, which is a time point at which DNA repair is already completed. Together, these findings indicate that WRAP53β is a novel player in the early ATM-, H2AX-, and MDC1-dependent accumulation of repair factors at sites of DNA damage.

### WRAP53β mediates recruitment of DNA repair proteins to DSBs through RNF8-mediated ubiquitylation

**Figure 2.**
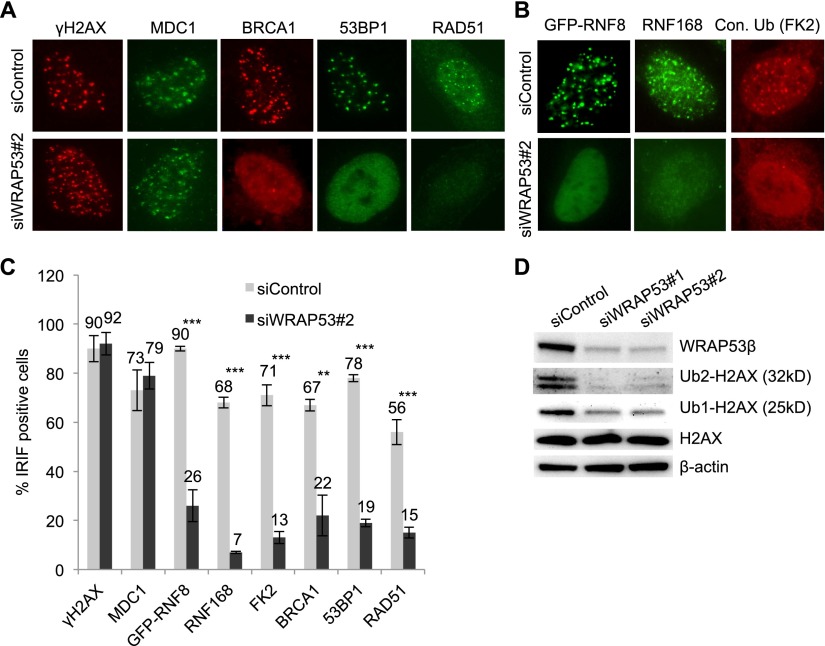
WRAP53β promotes recruitment of repair proteins to DSBs. (*A*) U2OS cells were transfected with siControl or siWRAP53#2 oligonucleotides for 24 h, exposed to IR (6 Gy) or left untreated, and, 1 h later, immunostained for γH2AX, MDC1, BRCA1, 53BP1, and RAD51. (*B*) U2OS cells treated as in *A* and then immunostained for RNF168 and conjugated ubiquitin (with the FK2 antibody). In the case of GFP-RNF8 staining, following treatment with oligonucleotides for 24 h, the cells were transiently transfected with the GFP-RNF8 plasmid for 8 h, exposed to IR (6 Gy), allowed to recover for 1 h, and then fixed and analyzed. (*C*) Quantification of the results in *A* and *B* as the percentage of 200 cells counted in each experiment whose nuclei contained IRIF. In the case of GFP-RNF8, only successfully transfected cells were counted. (*D*) U2OS cells were treated with the siRNAs indicated for 24 h, irradiated (6 Gy), allowed to recover for 1 h, and then subjected to Western blotting for WRAP53β, H2AX, and β-actin. The error bars depict the SEM. *n* = 3; (**) *P* < 0.01; (***) *P* < 0.001, as determined by Student’s *t*-test.

Having established that WRAP53β is involved in the DDR, we wanted to elucidate its mechanism of action in this process. Since WRAP53β is involved in intracellular trafficking, we asked whether WRAP53β could control recruitment of DNA repair proteins to DNA breaks. Interestingly, accumulation of BRCA1, 53BP1, and RAD51 at DSBs caused by IR was consistently impaired in cells depleted of WRAP53β, whereas the upstream DDR proteins MDC1 and γH2AX still formed foci (Fig. 2A). Introduction of siRNA-resistant Flag-WRAP53β into cells depleted of endogenous WRAP53β restored formation of DDR foci, which argues against off-targeting effects of the siRNA (Supplemental Fig. 2A–C).

Recruitment of BRCA1, 53BP1, and RAD51 to DSBs requires the ubiquitin ligase RNF8 (Huen et al. 2007; Mailand et al. 2007; Meerang et al. 2011), which we also confirmed here to be the case for RAD51 (Supplemental Fig. 2D). Indeed, depletion of WRAP53β impaired accumulation of both GFP-RNF8 and the E3 ligase RNF168, with which it works in close conjunction at DSBs (Fig. 2B; Doil et al. 2009). A similar reduction in the accumulation of HA-RNF8 at DSBs was observed in WRAP53β-depleted cells, whereas HA-MDC1 still formed foci (Supplemental Fig. 2E,F). This reduction of HA-RNF8 did not reflect any decrease in the levels of this protein (Supplemental Fig. 2G). In line with the reduction in the formation of both RNF8 and RNF168 foci, WRAP53β also reduced the total levels of ubiquitin conjugates at DSBs (Fig. 2B,C). The extent of this reduction was comparable with the loss of ubiquitylation caused by depletion of RNF8, RNF168, and MDC1 (Supplemental Fig. 2H). Furthermore, we examined RNF8- and RNF168-specific ubiquitylation of their substrate histone, H2AX, which is important for the recruitment of repair proteins to DSBs (Mailand et al. 2007). Indeed, WRAP53β knockdown reduced H2AX ubiquitylation (Fig. 2D). Together, these data show that WRAP53β is required for localization of RNF8 at DSBs and its subsequent ubiquitylation at these sites, thereby promoting assembly of DNA repair factors at DSBs.

### WRAP53β binds the forkhead-associated (FHA) domains of both RNF8 and MDC1

**Figure 3.**
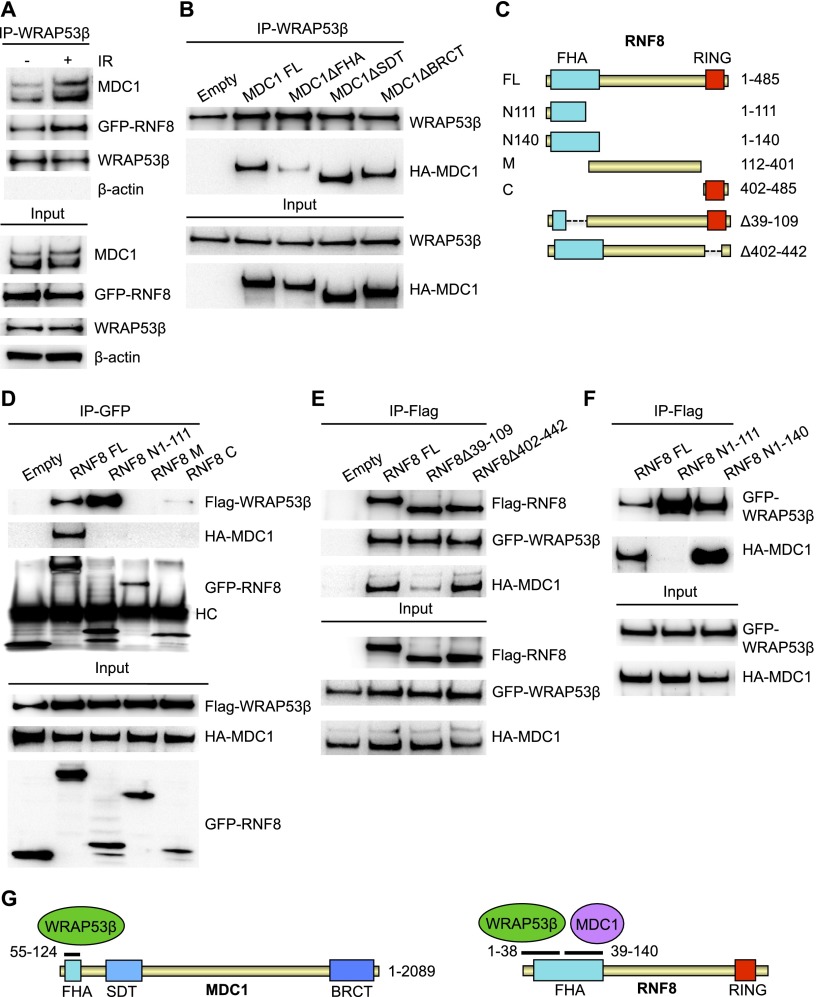
WRAP53β binds MDC1 and RNF8 via their FHA domains. (*A*) U2OS cells were either left untreated or irradiated with 6 Gy of IR, and, 30 min, later immunoprecipitation of WRAP53β was performed, followed by immunoblotting of WRAP53β, MDC1, GFP-RNF8, and β-actin. (*B*) U2OS cells were transfected with the indicated HA-MDC1 constructs for 16 h and irradiated with 2 Gy, and, 30 min later, immunoprecipitation of WRAP53β was performed, followed by immunoblotting of WRAP53β and HA-MDC1. (*C*) Schematic illustration of RNF8 deletion constructs. (*D*) U2OS cells were transiently transfected with EGFP-RNF8 plasmids, HA-MDC1, and Flag-WRAP53β for 16 h; irradiated; and subjected to immunoprecipitation of GFP followed by immunoblotting for GFP-RNF8, Flag-WRAP53β, and HA-MDC1. (HC) Heavy chain of the antibody. U2OS (*E*) and H1299 (*F*) cells were transiently transfected with Flag-RNF8 plasmids, HA-MDC1, and EGFP-WRAP53β for 16 h; irradiated; and subjected to Flag immunoprecipitation followed by immunoblotting for the indicated proteins. (*G*) Schematic illustration of the domain architecture of MDC1 and RNF8, where black lines mark WRAP53β- and MDC1-binding sites. Numbers indicate amino acids.

To obtain additional insight into how WRAP53β recruits RNF8 to DSBs, potential binding of WRAP53β to RNF8 and its upstream partner, MDC1, was explored (Huen et al. 2007; Kolas et al. 2007; Mailand et al. 2007). Indeed, WRAP53β was found to bind both MDC1 and RNF8 (Fig. 3A; Supplemental Fig. 3A,B) in a specific manner, as demonstrated by knockdown of WRAP53β (Supplemental Fig. 3C). These interactions were enhanced in response to DNA damage, although they were also present in nonirradiated cells (Fig. 3A). In addition, WRAP53β and RNF8 clearly colocalized at sites of DNA damage (Supplemental Fig. 3D). To further investigate the binding of WRAP53β to MDC1 and RNF8, a series of MDC1 internal deletion mutants were tested for binding to WRAP53β (Wang et al. 2011). This revealed that WRAP53β binds the phosphopeptide recognition FHA domain of MDC1 (amino acids 55–124), since deletion of this domain abolished their interaction, while deletion of the SDT repeats (amino acids 200–429) or the tandem BRCT domain (amino acids 1893–2089) of MDC1 did not alter binding to WRAP53β (Fig. 3B).

When mapping RNF8, we found that WRAP53β bind to its N terminus (containing the FHA domain) but not the middle part or C terminus of this protein (containing the RING domain) (Fig. 3C,D). Further deletion mapping identified a small section in the extreme N terminus of RNF8 (amino acids 1–38) critical for WRAP53β binding, since an internal deletion mutant of RNF8 lacking amino acids 39–109 retained the ability to bind WRAP53β (Fig. 3E).

The FHA domain of RNF8 is located between amino acids 13 and 140, encompassing an N-terminal phosphopeptide-binding part and an α-helical extension (amino acids 130–140) that is located away from the phosphopeptide-interacting surface (Huen et al. 2007; Orthwein et al. 2014). A shorter variant of the RNF8 FHA (amino acids 17–111) has also been predicted (Mailand et al. 2007; Luijsterburg et al. 2012). We next explored whether WRAP53β and MDC1, which bind the FHA domain of RNF8 in a phosphorylation-dependent manner, target different regions of this domain. Indeed, while an N-terminal version of RNF8 containing amino acids 1–140 was sufficient in binding both MDC1 and WRAP53β, a shorter N-terminal variant of RNF8 (amino acids 1–111) bound only WRAP53β, not MDC1 (Fig. 3D,F). Since deletion of amino acids 39–109 of RNF8 does not alter binding to WRAP53β but impairs binding to MDC1 (Fig. 3E), we conclude that the beginning of the RNF8 FHA domain (amino acids 1–38) mediates binding to WRAP53β, while the end of the RNF8 FHA domain (amino acids 39–140) is involved in binding MDC1 (Fig. 3G). Together, these data indicate that the FHA domains of both RNF8 and MDC1 mediate binding to WRAP53β and that these regions bound by WRAP53β are not involved in the direct interaction between RNF8 and MDC1.

### WRAP53β controls the interaction between RNF8 and phosphorylated MDC1

**Figure 4.**
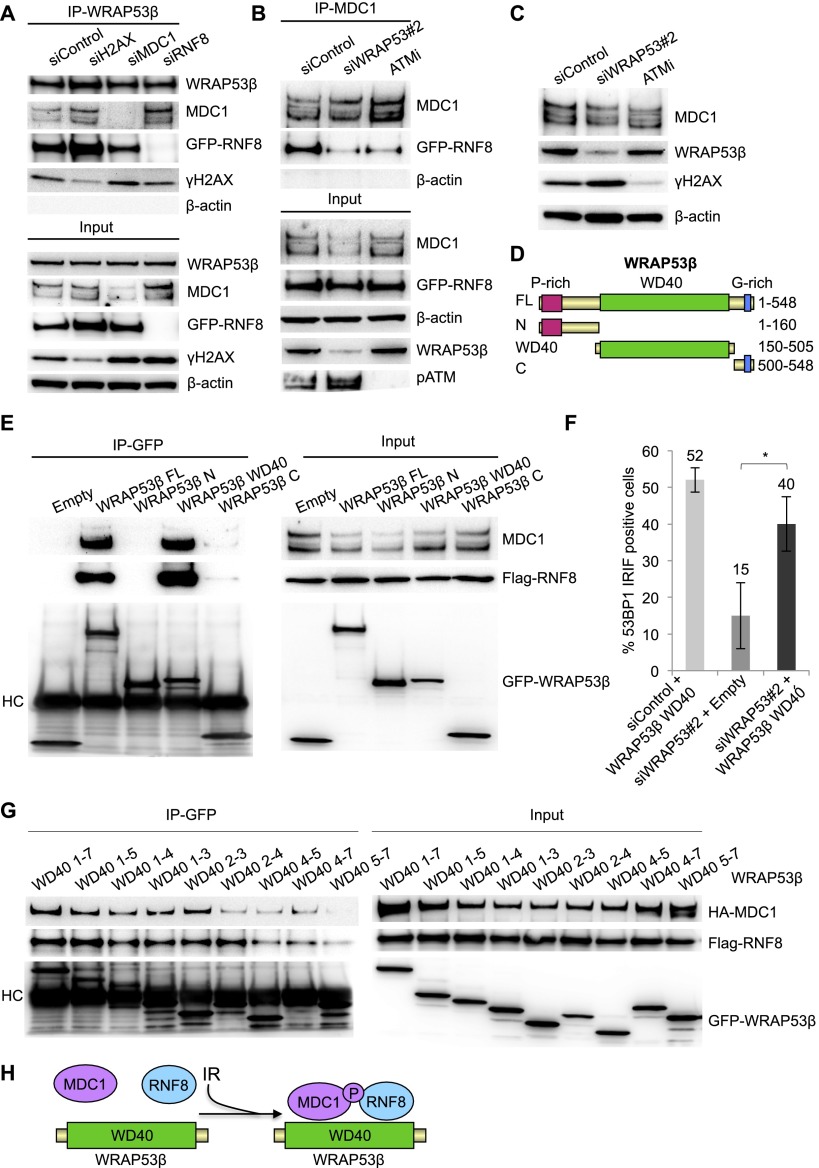
WRAP53β facilitates MDC1–RNF8 interaction through its WD40 domain. (*A*) U2OS cells were treated with the siRNAs indicated for 48 h and with GFP-RNF8 for 24 h (all samples), irradiated with 6 Gy, and, 30-min later, subjected to immunoprecipitation of WRAP53β followed by immunoblotting of WRAP53β, MDC1, RNF8, γH2AX, and β-actin. (*B*) Immunoprecipitation of MDC1 in irradiated (6 Gy, 15-min recovery) U2OS cells treated with the siRNA indicated for 48 h or ATM inhibitor (ATMi) for 24 h. All samples were transfected with GFP-RNF8 for 16 h. (*C*) U2OS cells were treated with the siRNAs indicated for 48 h or ATM inhibitor for 16 h, irradiated with 6 Gy, allowed to recover for 15 min, and then subjected to Western blotting of MDC1, WRAP53β, γH2AX, and β-actin. (*D*) Schematic illustration of EGFP-tagged deletion constructs of WRAP53β. (*E*) U2OS cells were transiently transfected with the indicated EGFP-WRAP53β plasmids and Flag-RNF8 for 16 h, irradiated, and subjected to GFP immunoprecipitation followed by immunoblotting for MDC1, Flag-RNF8, and GFP-WRAP53β. (HC) Heavy chain of the antibody. (*F*) U2OS cells were transfected with siControl or siWRAP53#2 oligonucleotides for 8 h followed by transfection of EGFP-Empty or EGFP-WRAP53β WD40 (1–7) for 16 h, exposed to IR (6 Gy), and, after 1 h, immunostained for 53BP1 followed by quantification of the results. The graph in *A* shows the percentage of 100 GFP transfected cells in each experiment whose nuclei were 53BP1-positive. The error bars depict the SEM. *n* = 3; (*) *P* < 0.05, as determined by Student’s *t*-test. (*G*) U2OS cells were transiently transfected with the indicated EGFP-WRAP53β plasmids, HA-MDC1, and Flag-RNF8 for 16 h; irradiated; and subjected to immunoprecipitation of GFP followed by immunoblotting for HA-MDC1, Flag-RNF8, and GFP-WRAP53β. (*H*) Schematic illustration of how WRAP53β scaffolds the MDC1–RNF8 complex. Upon DNA damage, WRAP53β binds MDC1 and RNF8 simultaneously via its WD40 domain and facilitates their interaction.

To explore whether the scaffold protein WRAP53β regulates complex formation between RNF8 and MDC1, we first examined the organization of interactions between WRAP53β, MDC1, RNF8, and γH2AX. Knockdown H2AX, MDC1, and RNF8 and subsequent immunoprecipitation of WRAP53β revealed that interaction of WRAP53β with MDC1 is independent of RNF8 and H2AX, and interaction of WRAP53β with RNF8 is independent of MDC1 and H2AX (Fig. 4A). However, the interaction between MDC1 and RNF8 was clearly attenuated in irradiated cells depleted of WRAP53β (Fig. 4B).

Interaction between RNF8 and MDC1 is dependent on phosphorylation of MDC1 by ATM (Huen et al. 2007; Kolas et al. 2007; Mailand et al. 2007). To characterize the manner by which WRAP53β facilitates these interactions, we first examined whether WRAP53β affects phosphorylation of MDC1. Phosphorylated MDC1 migrated more slowly on an SDS gel than the unphosphorylated form (Stewart et al. 2003; Zhang et al. 2005), and preventing this phosphorylation by inhibition of ATM thus changed the mobility of the MDC1 band. In contrast, no such shift was observed when WRAP53β was knocked down (Fig. 4C), suggesting that phosphorylation of MDC1 by ATM occurs independently of WRAP53β and, moreover, does not account for the loss of RNF8–MDC1 interaction in WRAP53β-depleted cells.

To explore whether the WD40 domain of WRAP53β mediates MDC1–RNF8 interaction, a series of EGFP-WRAP53β deletion constructs were generated (Fig. 4D). Indeed, the WD40 domain of WRAP53β proved to be both necessary and sufficient for binding both MDC1 and RNF8, whereas the N or C terminus alone was unable to bind either of these factors (Fig. 4E). Moreover, introduction of the WD40 domain into cells transfected with WRAP53β siRNA restored 53BP1 focus formation, confirming that the WD40 domain of WRAP53β is critical for RNF8-mediated DNA DSB repair (Fig. 4F).

We further divided the WD40 domain of WRAP53β into different combinations of repeats (Supplemental Fig. 4A). Using these constructs, MDC1 and RNF8 were found to preferentially bind repeats 2 and 3 (Fig. 4G).

Missense mutations in WD40 repeats 1, 5, and 6 of WRAP53β cause the cancer predisposition syndrome dyskeratosis congenita and associate with nuclear exclusion and reduced expression of this protein (Zhong et al. 2011). These mutations were individually introduced into a vector encoding EGFP-WRAP53β, and immunofluorescence confirmed a markedly reduced nuclear localization of these disease variants of WRAP53β (Supplemental Fig. 4A–C). Nevertheless, all mutants retained the ability to bind MDC1 and RNF8 (Supplemental Fig. 4D). Although these mutations localize outside of repeats 2 and 3, involved in binding MDC1 and RNF8, such WRAP53β mutants are excluded from the nucleus where MDC1 and RNF8 exert their function, indicating that interaction between these proteins likely is impaired in WRAP53β-associated dyskeratosis congenita. Taken together, these findings demonstrate that, in response to DNA damage, WRAP53β binds MDC1 and RNF8 simultaneously via its WD40 domain, thereby facilitating interaction between these proteins and accumulation of RNF8 at DSBs (Fig. 4H).

### WRAP53β promotes HR and nonhomologous end-joining (NHEJ) repair

**Figure 5.**
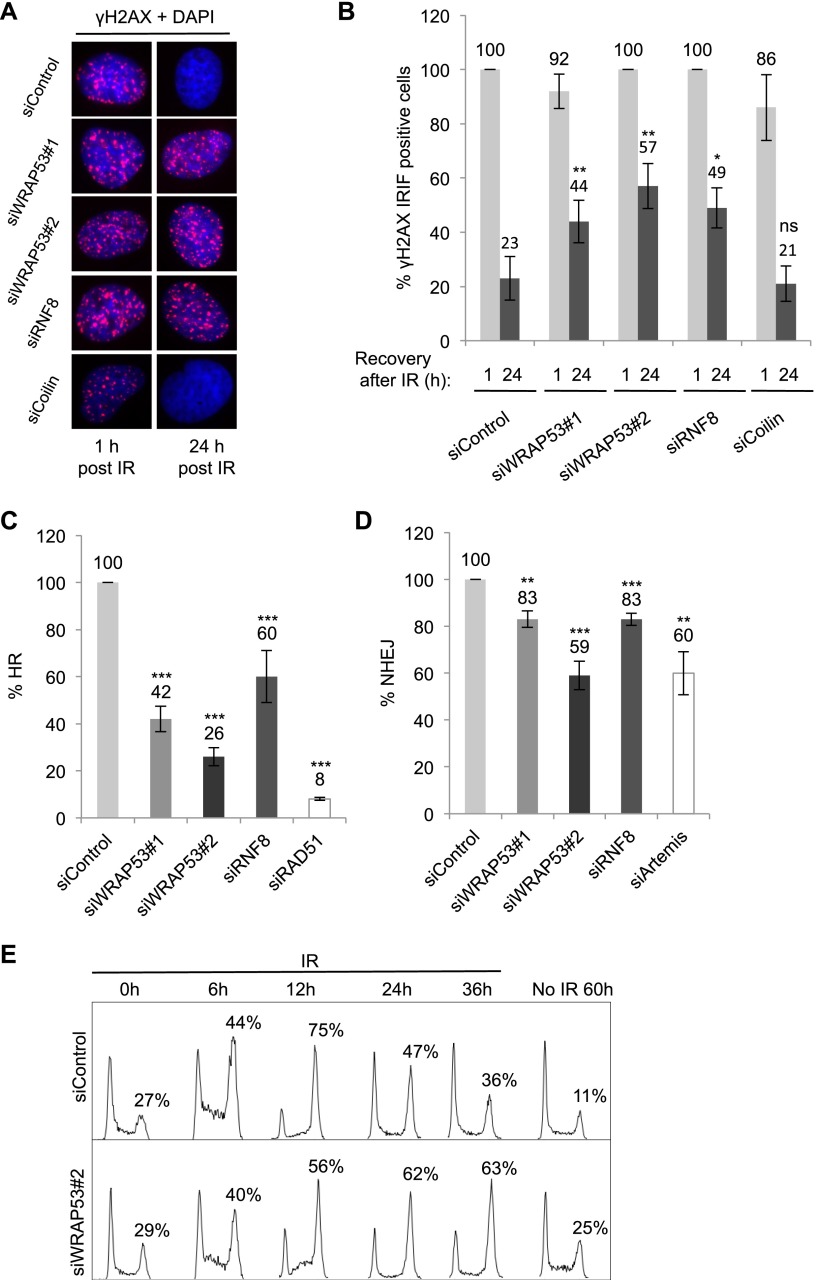
WRAP53β promotes HR and NHEJ. (*A*) U2OS cells were treated with the siRNAs indicated for 24 h, exposed to 6 Gy of IR, fixed 1 h or 24 h later, and immunostained for γH2AX. (*B*) Quantification of the results in *A* showing the percentage of nuclei containing >10 γH2AX foci (*n* = 200). (*C*,*D*) HR (*C*) and NHEJ (*D*) efficiency following treatment of the cells with the siRNA indicated for 48 h. DR-GFP (HR) and EJ5-GFP (NHEJ) reporter systems were used in the FACS analysis, with siRAD51 and siArtemis as positive controls. (*E*) Cells were transfected with siRNA for 24 h, exposed to IR (3 Gy), harvested at the time points indicated, and subjected to flow cytometry. Nonirradiated cells were treated with siRNA alone for 60 h. The error bars depict the SEM. *n* = 3; (*) *P* < 0.05; (**) *P* < 0.01; (***) *P* < 0.001, as determined by Student’s *t*-test.

Next, the impact of WRAP53β on repair of DSBs was evaluated by examining the clearance of γH2AX IR-induced foci (IRIF). Control cells showed a rapid induction of γH2AX foci following exposure to IR, and these foci were completely resolved 24 h after IR, indicating efficient DNA repair. Cells lacking WRAP53β showed the same rapid induction of γH2AX foci 1 h after IR; however, 24 h after IR, a significant amount of residual γH2AX foci still remained (Fig. 5A,B), indicating defective repair. RNF8 depletion affected residual γH2AX foci in the same manner as WRAP53β deficiency (Fig. 5A,B). Western blotting confirmed elevated γH2AX levels in cells depleted of WRAP53β 24 h after IR (Supplemental Fig. 5A). Persisting foci following WRAP53β depletion were not linked to its known interaction partner, coilin, since knockdown of coilin did not influence the formation of γH2AX foci either with or without IR (Fig. 5A,B; Supplemental Fig. 5B; data not shown). Moreover, since U2OS cells lack telomerase, the involvement of WRAP53β in DNA repair appears to be independent of this enzyme. Indirect effects on apoptosis were also excluded (Supplemental Fig. 5C). In addition, pulse-field gel electrophoresis demonstrated that stimulation of H2AX phosphorylation by IR of WRAP53β-depleted cells was due to accumulation of DNA breaks (Supplemental Fig. 5D). To determine whether depletion of WRAP53β renders cells sensitive to IR, clonogenic survival assays were performed. However, since prolonged silencing of WRAP53β triggered massive apoptosis and a 80%–95% reduction in colony formation in nonirradiated H1299 and U2OS cells (data not shown), these experiments were inconclusive.

To assess the involvement of WRAP53β in DSB repair, we used GFP reporter assays for the HR and NHEJ repair pathways. The HR assay was based on U2OS cells carrying the DR-GFP construct, in which expression of exogenous I-SceI introduces a single DSB. Repair of this break by HR creates a functional GFP gene, and its expression level is an accurate readout for HR efficiency (Pierce et al. 1999). Strikingly, this revealed that WRAP53β knockdown lowered HR by 74%, which is comparable with the 92% reduction obtained upon depleting RAD51, a known component of HR. RNF8 silencing resulted in a 40% reduction in the efficiency of HR (Fig. 5C). The NHEJ assay worked in a fashion similar to the HR assay but used a GFP reporter (EJ5-GFP) with two I-SceI sites flanking a *puro* gene. Following I-SceI cleavage, the *puro* gene was removed, and repair of these DSBs by NHEJ placed a promoter adjacent to the GFP gene and allowed expression of GFP (Gunn and Stark 2012). We found that NHEJ repair was attenuated 41% and 17% by knockdown of WRAP53β and RNF8, respectively (Fig. 5D). Depletion of Artemis, a key component of NHEJ, decreased this repair by 40%. Notably, depletion of WRAP53β had a more profound effect on HR and NHEJ compared with knockdown of RNF8. Moreover, codepletion of WRAP53β and RNF8 did not further impair HR or NHEJ repair as compared with knocking down WRAP53β alone (Supplemental Fig. 5E). This suggests that RNF8 acts in the same pathway as WRAP53β, while WRAP53β plays additional roles in DNA DSB repair.

To gain further insight into WRAP53β-mediated DDR effects, we studied cell cycle progression in WRAP53β-depleted cells in combination with IR. In the presence of DNA damage, the cell cycle is delayed to allow more time for repair. WRAP53β-depleted cells exposed to IR demonstrated normal G2/M checkpoint activation but a prolonged G2/M arrest, suggesting delayed recovery due to defective DNA repair (Fig. 5E). Thus, WRAP53β plays a key role in both HR and NHEJ repair of DSBs, and, in its absence, DNA breaks accumulate, and checkpoint activation persists.

### WRAP53β protects cells against spontaneous DNA damage

**Figure 6.**
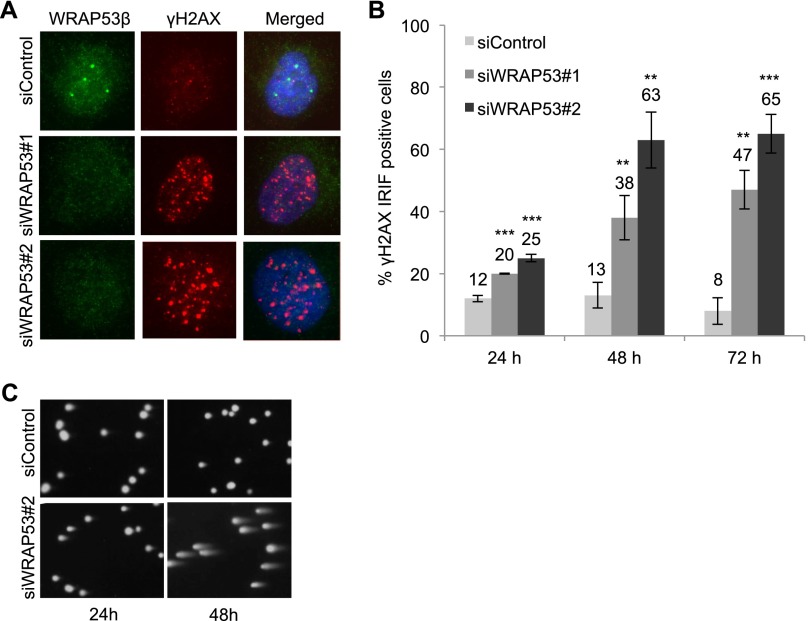
WRAP53β protects cells against accumulation of spontaneous DNA damage. (*A*) U2OS cells were treated with siControl or two different siWRAP53 oligonucleotides (siWRAP53#1 and siWRAP53#2) for 24 h, fixed, and immunostained for WRAP53β and γH2AX. (*B*) The percentage of nuclei in *A* containing >10 γH2AX foci was quantified in the 200 cells counted for each experiment. (*C*) After treating U2OS with siWRAP53#2 or siControl for 24 h or 48 h, DNA damage was assessed by the alkaline comet assay. The error bars depict the SEM. *n* = 3; (**) *P* < 0.01; (***) *P* < 0.001, as determined by Student’s *t*-test.

We observed that siRNA-mediated knockdown of WRAP53β in U2OS cells elevated the number of γH2AX foci in nonirradiated cells within 24 h, indicating accumulation of spontaneous DNA damage (Fig. 6A,B). Moreover, depletion of RNF8 elevated spontaneous formation of γH2AX foci in a similar manner (Supplemental Fig. 6A). Prolonged silencing of WRAP53β (48–72 h) was associated with more spontaneous γH2AX foci and apoptosis apparent from 72 h after siRNA treatment (Fig. 6B; Supplemental Fig. 6B). Western blotting of γH2AX levels confirmed its increased phosphorylation following WRAP53β depletion (Supplemental Fig. 6C). A similar phenomenon was seen in H1299 and HeLa cells (Supplemental Fig. 6D–F). Both alkaline and neutral comet assays revealed that cells lacking WRAP53β contain larger numbers of sporadic DNA breaks (Fig. 6C; Supplemental Fig. 6G). Thus, we conclude that WRAP53β protects cells against spontaneous DNA damage. The fact that WRAP53β knockdown triggers spontaneous activation of γH2AX in H1299 and HeLa cells, which lack and have inactivated p53, respectively, demonstrates that the role played by WRAP53β in the protection against spontaneous DNA damage is independent of WRAP53α-mediated regulation of p53. Taken together with our other findings, these results suggest that WRAP53β plays an essential role in the DDR and that attenuation of this function disturbs repair of DSBs and leads to accumulation of spontaneous DNA breaks, which are considered to be the major factor underlying genomic instability.

## Discussion

**Figure 7.**
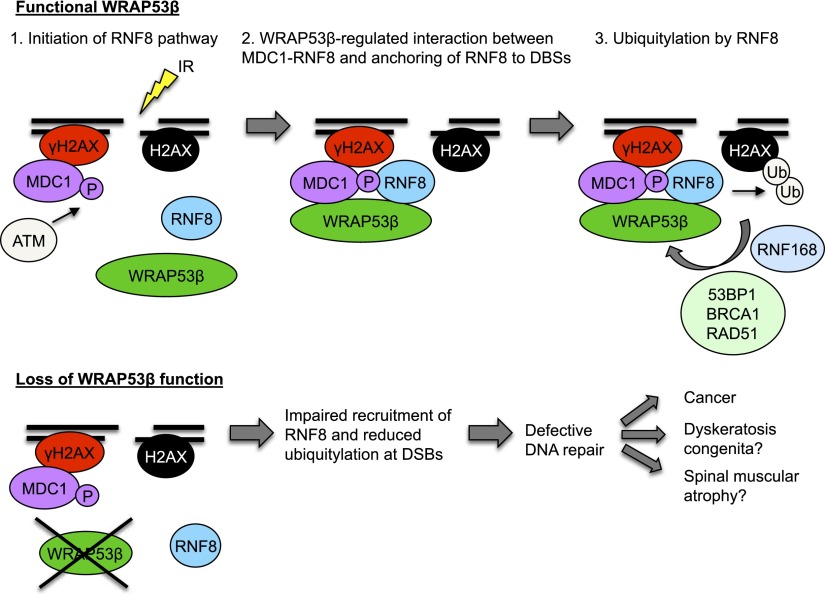
Schematic model of WRAP53β function in the DDR pathway. (Step 1) In response to IR, γH2AX and MDC1 accumulate at DSBs independently of WRAP53β. ATM-mediated phosphorylation of MDC1 makes MDC1 competent to bind RNF8. However, RNF8 is not yet localized at DSBs. (Step 2) WRAP53β is recruited to sites of DNA damage in an ATM-, H2AX-, and MDC1-dependent manner. Simultaneous binding of MDC1 and RNF8 to WRAP53β facilitates their direct interaction and retention of RNF8 at DSBs. (Step 3) Once assembled at DSBs, RNF8 catalyzes ubiquitylation of H2AX. Ubiquitylation at DSBs enables recruitment and accumulation of 53BP1, BRCA1, and RAD51 and subsequent DNA repair.

The present investigation identifies the scaffold protein WRAP53β as a novel regulator of DSB repair that orchestrates the assembly of repair factors at such sites of DNA damage. In response to IR, WRAP53β is recruited to DNA breaks in an ATM-, H2AX-, and MDC1-dependent manner. Our findings reveal that WRAP53β targets the critical ubiquitin ligase RNF8 to DNA lesions by promoting MDC1–RNF8 interactions. Accordingly, WRAP53β is required for efficient ubiquitylation of damaged chromatin, an important aspect of the signaling and repair of DNA damage (Fig. 7). Consequently, loss of WRAP53β disrupts DNA repair by both HR and NHEJ and leads to accumulation of DSBs, consistent with attenuated accumulation and activity of RNF8 at these breaks (Meerang et al. 2011).

In the DDR recruitment cascade, RNF8 acts immediately downstream from MDC1. ATM-mediated phosphorylation of MDC1 generates binding sites recognized by the FHA domain of RNF8, which is critical for the targeting of RNF8 to DNA breaks (Huen et al. 2007; Kolas et al. 2007; Mailand et al. 2007). In agreement with previous reports, we found that localization of RNF8 to DSBs is dependent on its interaction with MDC1. In WRAP53β-depleted cells, IR did not induce the formation of RNF8 foci, and, indeed, interaction between RNF8 and MDC1 was disrupted. The loss of binding between RNF8 and MDC1 was not due to abrogated MDC1 phosphorylation, since hyperphosphorylation of MDC1 was observed even when WRAP53β was knocked down. Moreover, assembly of MDC1 at DSBs was normal in WRAP53β-depleted cells. Instead, our present observations suggest that WRAP53β promotes the accumulation of RNF8 to sites of DNA damage by tethering MDC1 and RNF8 together. WRAP53β contains a WD40 domain, a highly conserved and well-established domain for protein interactions. We show here that in response to DNA damage, simultaneous binding of MDC1 and RNF8 to this region is required for their interaction. In this respect, WRAP53β appears to function as a scaffold for stable MDC1–RNF8 complex formation, thereby supporting the formation of RNF8 IRIF and subsequent ubiquitin-dependent assembly of downstream repair factors. Thus, the proper accumulation and functioning of RNF8 at DNA DSBs appear to be regulated via both phosphorylation of MDC1, which enables direct binding between MDC1 and RNF8, and WRAP53β, which scaffolds and stabilizes the MDC1–RNF8 interaction. Similarly, we demonstrated previously that WRAP53β controls assembly of the survival of motor neuron (SMN) protein in Cajal bodies by mediating the direct interaction between SMN and the coilin protein (Mahmoudi et al. 2010).

This role of WRAP53β in the DDR bears striking similarities to the function of another WD40 protein, PALB2. In response to DNA damage, PALB2 facilitates interaction between BRCA1 and BRCA2 and the subsequent anchoring of BRCA2 to sites of DNA damage (Xia et al. 2006). Although PALB2 binds both of these proteins even in the absence of DNA damage, it promotes their interaction and localization of BRCA2 to DSBs only after the induction of DNA damage by IR, whereas BRCA1 localizes to DSBs independently of PALB2, where it helps recruit PALB2 to these sites (Zhang et al. 2009). In the same manner, WRAP53β facilitates MDC1 and RNF8 interaction, controlling the accumulation of RNF8 only at DSBs, whereas MDC1 form IRIF independently of WRAP53β and help recruit WRAP53β to DSBs.

We show that WRAP53β binds the FHA domains of both MDC1 and RNF8, a domain known to primarily mediate phosphorylation-dependent protein interactions with a high specificity toward phosphorylated threonines. However, phosphorylation-independent interactions mediated by FHA domains have also been described. The FHA domain of MDC1 has been shown to bind Thr68-phosphorylated Chk2, Ser1981-phosphorylated ATM, and unphosphorylated RAD51 (Coster and Goldberg 2010), while the same domain of RNF8 binds phosphorylated MDC1 and CHD4 independent of phosphorylation (Huen et al. 2007; Kolas et al. 2007; Mailand et al. 2007; Luijsterburg et al. 2012). The phosphorylation-independent interaction between RNF8-FHA and CHD4 excludes the α-helical extension of RNF8 (amino acids 130–140) (Luijsterburg et al. 2012). Similarly, mutants of RNF8 either lacking its α-helical extension (N1–111) or with an inactivated FHA domain (Δ39–109) still bound WRAP53β, suggesting that WRAP53β also interacts with the RNF8 FHA domain independently of phosphorylation and the α-helical extension. We also show that WRAP53β targets the extreme N terminus of RNF8-FHA, while MDC1 binds to downstream parts of this same domain, enabling their simultaneous interaction with RNF8. Thus, our present investigation suggests that the FHA domains of MDC1 and RNF8 target sites within the WD40 domain of WRAP53β, but whether this requires phosphorylation of WRAP53β remains to be investigated.

Many DDR proteins, especially those involved in the initial steps in the DSB signaling cascade, also participate in telomere maintenance (d’Adda di Fagagna et al. 2004). Our current study establishes that WRAP53β also both acts at telomeres and is involved in the DDR pathway. This raises the interesting possibility that WRAP53β might regulate telomere elongation and DNA repair via a common mechanism. Indeed, it is known that WRAP53β maintains telomeres by recruiting the telomerase enzyme to telomeres (Venteicher et al. 2009). However, the role of WRAP53β in DNA repair must be independent of telomerase and distinct from its involvement in telomere maintenance, since the U2OS cells characterized here do not express telomerase but instead maintain their telomeres by alternative lengthening of telomeres (ALT). Moreover, depletion of coilin, which interacts with WRAP53β, did not disrupt DNA repair in the same manner as knocking down WRAP53β itself. Since coilin has previously been implicated in NHEJ repair (Velma et al. 2010), WRAP53β may act upstream of coilin, which would explain why its loss results in more extensive DDR defects.

We observed that knockdown of WRAP53β consistently enhanced the level of spontaneous DNA breaks. HR eliminates such lesions formed during the process of DNA replication. Accordingly, cells deficient in proteins involved in this repair mechanism, such as BRCA1, exhibit high levels of sporadic DNA damage (Venkitaraman 2002; Saleh-Gohari et al. 2005; Petermann and Helleday 2010). These considerations are consistent with the involvement of WRAP53β in HR and also suggest that reduced expression of this protein may contribute to genomic instability and carcinogenesis. Consistent with this hypothesis, we found that lack or low levels of WRAP53β correlated with reduced patient survival and attenuated DDR in ∼400 ovarian cancer patients (data not shown). This indicates that loss of WRAP53β-mediated DNA repair contributes to the formation and/or progression of tumors.

Germline mutations in WRAP53β result in dyskeratosis congenita, which is characterized by premature aging and a predisposition to develop cancer. Such mutations patently reduce nuclear expression of WRAP53β and lead to progressive shortening of telomeres due to impaired WRAP53β-mediated trafficking of telomerase in cells from these patients (Zhong et al. 2011). Similarly, patients with spinal muscular atrophy, a neurodegenerative disorder, exhibit functional loss of nuclear WRAP53β. Our discovery that WRAP53β is a key component of the DDR suggests that defects in DNA repair may also contribute to the manifestations of these diseases, although this remains to be shown.

In summary, the present investigation establishes WRAP53β as a novel regulator of DSB repair, serving as a platform for the formation of protein complexes involved in the DDR. Indeed, our findings highlight the role of scaffold proteins in various aspects of this response. Moreover, in light of its additional participation in telomerase trafficking, WRAP53β appears to play a major role in guiding enzymes responsible for the restoration and maintenance of genome integrity. Our model suggests a potentially important novel mechanism underlying the defects in DNA repair associated with neurodegeneration, aging, and cancer and may thus have far-reaching clinical and therapeutic implications.

## Materials and methods

### Cells and culture conditions

U2OS cells were maintained in McCoy’s 5A medium (HyClone, Thermo Scientific); fibroblasts were kept in minimum essential medium (HyClone, Thermo Scientific); and U2OS (DR-GFP and EJ5-GFP), H1299, and HeLa cells were maintained in Dulbecco’s modified Eagle medium (DMEM) (HyClone, Thermo Scientific) supplemented with 10% fetal bovine serum (FBS) (HyClone) and 2.5 µg/mL plasmocin (InvivoGen) at 37°C in 5% CO_2_ humidified incubators. MCF7 cells stably expressing ddI-PpoI were grown in DMEM supplemented with 10% FBS + 1 μg/mL puromycin (Sigma). 4-OHT (Sigma) was added to a final concentration of 1 μM. Shield-1 (Cheminpharma) was added to a final concentration of 1 μM for 3 h to stabilize the ddI-PpoI fusion protein. MCF7 cells were arrested in G1 phase of the cell cycle, in which NHEJ should be the predominant DSB repair mechanism, by cultivating them in medium containing 0.1% FBS for 24 h before DSB induction.

### Laser microirradiation

Localized DNA damage was generated by exposure of cells to a UV-A laser. Fibroblasts and U2OS and H1299 cells were presensitized with 10 mM 5-bromo-2′-deoxyuridine (BrDU) for 24 h at 37°C. Prior to microscopy, the medium was replaced for a phenol red-free medium. Microirradiation was performed with a confocal microscope equipped with a 365-nm UV-A laser.

### IR

γ-Irradiation was performed with a ^137^Cs source (Scanditronix) at the Karolinska Institutet, Stockholm, at a photon dose rate of 0.5 Gy/min. Dosimetry was done with an ionization chamber as well as with ferro-sulphate.

### Immunofluorescence microscopy

Cells were grown on sterilized coverslips and fixed with 4% paraformaldehyde (PFA) for 15 min at room temperature. They were then permeabilized with 0.1% Triton X-100 for 5 min at room temperature followed by 30 min of blocking in blocking buffer (2% BSA, 5% glycerol, 0.2% Tween20, 0.1% NaN_3_). Coverslips were subsequently incubated for 1 h in primary antibody and 40 min in secondary antibody diluted in blocking buffer. The coverslips were mounted with VectaShield mounting medium with DAPI (Vector Laboratories). Images were acquired with a Zeiss Axioplan 2 microscope equipped with an AxioCam HRm Camera using a 40× or 63× oil immersion lens and processed using Axiovision release 4.7.

#### Pre-extraction

To visualize WRAP53β IRIF, the cells were first washed with PBS; incubated for 3 min at room temperature with cytoskeleton (CSK) buffer containing 10 mM pipes (pH 7.0), 100 mM NaCl, 300 mM sucrose, 3 mM MgCl_2_, and 0.7% Triton X-100; and thereafter incubated for another 3 min with the same CSK buffer supplemented with 0.3 mg/mL RNase A (CSK+R). After these treatments, the cells were washed once again with PBS and then fixed in 4% PFA.

### Antibodies

The WRAP53β antibodies used were rabbit α-WRAP53-C2 (used for Western blot, immunoprecipitation, ChIP, and immunofluorescence experiments; Innovagen AB, catalog no. PA-2020-100), rabbit α-WDR79 (used for immunofluorescence; Bethyl Laboratories, catalog no. A301-442A-1), rabbit α-WRAP53 (used for immunofluorescence; Proteintech, catalog no. 14761-1-AP), rabbit α-WDR79 (used for immunofluorescence; Abnova, catalog no. H00055135-D01P), mouse monoclonal α-WDR79 (clone 1F12; used for immunofluorescence; Abnova, catalog no. H00055135-M04), and mouse polyclonal α-WDR79 (used for immunofluorescence; Abnova, catalog no. H00055135-B01P).

The following antibodies were used in immunoprecipitation, immunofluorescence, and Western blots: mouse α-γH2AX (Millipore, catalog no. 05-636), rabbit α-γH2AX (Cell Signaling, catalog no. 2577), rabbit α-H2AX (Abcam, catalog no. ab11175), rabbit α-MDC1 (Abcam, catalog no. ab11169), mouse α-MDC1 (Abcam, catalog no. ab50003), mouse α-pATM (Santa Cruz Biotechnology, catalog no. sc-47739), mouse α-RNF8 (Santa Cruz Biotechnology, catalog no. sc-271462), rabbit α-RNF8 (provided by Michael Huen), rabbit α-RNF168 (Millipore, catalog no. ABE367), mouse α-ubiquitin (FK2; Calbiochem, catalog no. ST1200), rabbit α-53BP1 (Novus Biologicals, catalog no. NB100-904), mouse α-BRCA1 (Santa Cruz Biotechnology, catalog no. sc-6954), rabbit α-RAD51 (Santa Cruz Biotechnology, catalog no. sc-8349), rabbit α-coilin (Santa Cruz Biotechnology, catalog no. sc-32860), mouse α-β-actin (Sigma), rabbit α-GFP (Abcam, catalog no. ab290), mouse α-Flag (Agilent Technologies, catalog no. 200472-21), normal rabbit IgG (Santa Cruz Biotechnology, catalog no. sc-2027), and normal mouse IgG (Santa Cruz Biotechnology, catalog no. sc-2025). The secondary antibodies used were sheep α-mouse HRP (GE Healthcare, catalog no. NA931V), donkey α-rabbit HRP (GE Healthcare, catalog no. NA934V), goat α-rabbit HRP (Cell Signal, catalog no. 7074), horse α-mouse HRP (Cell Signal, catalog no. 7076), goat α-rabbit Alexa Fluor 488 (Invitrogen, catalog no. A11008), goat α-mouse Alexa Fluor 488 (Invitrogen, catalog no. A11029), and donkey α-mouse Alexa Fluor 594 (Invitrogen, catalog no. A21203).

### siRNA transfections

The siRNA oligonucleotides used were siWRAP53#1 (Qiagen, catalog no. SI00388941), siWRAP53#2 (Qiagen, catalog no. SI00388948), siH2AX (Qiagen, catalog no. SI00032844), siMDC1 (Dharmacon, catalog no. L-003506-00-0005), siRNF8 (Dharmacon, catalog no. L-006900-00-0005), siRAD51 (Qiagen, catalog no. SI02663682), siCoilin (Qiagen, catalog no. SI00350343), siArtemis (Qiagen, catalog no. SI00133945), and siControl (Qiagen, catalog no. 1027280). siRNA (10–20 nM) was transfected into cells using HiPerfect (Qiagen) transfection reagent in accordance with the supplier’s recommendations.

### Treatment with small-molecule inhibitors

ATM (KU55933) and DNA-PK (NU7441) inhibitors were obtained from TOCRIS bioscience. The ATR inhibitor (VE-821) was obtained from Axon MedChem (catalog no. Axon 1893). Where appropriate, 10 μM ATM inhibitor, 2 μM DNA-PK inhibitor, and 2.5 μM ATR inhibitor were added to the culture medium 6–24 h prior to IR.

### ChIP

The ChIP protocol was adapted for use with magnetic beads (Dynabeads M-280 sheep anti-rabbit, Invitrogen). MCF7 cells expressing ddI-PpoI were treated with Shield-1 for 3 h followed by 4-OHT and were collected for ChIP. Immunoprecipitations were first washed in low-salt wash (150 mM NaCl, 0.1% SDS, 1% Triton X-100, 2 mM EDTA, 20 mM Tris-HCl), followed by high-salt wash (500 mM NaCl, 0.1% SDS, 1% Triton X-100, 2 mM EDTA, 20 mM Tris-HCl), followed by three washes in LiCl wash (0.25 M LiCl, 1% IGEPAL**, 1% deoxycholic acid, 1% IGEPAL, 10 mM Tris-HCl). Washes were performed by rotation for 5 min at room temperature. An appropriate negative control using normal rabbit IgG was performed for the ChIP experiment.

### Plasmids

Plasmid transfections were performed using Lipofectamine 2000 reagent (Life Technologies) according to the manufacturer’s recommendations. To generate EGFP or 3xFlag-tagged WRAP53β and RNF8 constructs, inserts were amplified by PCR (Advantage 2 polymerase, Clontech) and subcloned into pEGFP-C1 (Clontech) or p3XFlag-CMV10 (Sigma, catalog no. E7658) expression vectors. All primers used for PCR amplifications are listed in Supplemental Table 1.

### Statistical analysis

The analyses were performed using Microsoft Excel 2011. Two-tailed Student’s *t*-test was used to determine statistical significance.

### Western blotting

For cell extracts for Western blot analysis, cells were harvested, washed, and lysed in ice-cold Western blot lysis buffer (100 mM Tris-HCL at pH 8, 150 mM NaCl, 1% NP-40, 1% PMSF, 1% protease inhibitor cocktail) for 30 min on ice. Lysates were centrifuged at 14,000 rpm for 15 min at 4°C, and protein concentrations were determined using Bradford assay (Bio-Rad). Western blot was performed according to standard procedures.

### Immunoprecipitation

Cells were lysed in NP40 buffer (150 mM NaCl, 50 mM Tris-HCL at pH 8,0, 1% NP40, 1% protease inhibitor cocktail) for 15 min on ice followed by three 5-sec sonications. Protein lysates were spun down at 6000 rpm for 5 min, and protein concentrations were quantified by Bradford assay (Bio-Rad). Proteins were immunoprecipitated with 1 µg of antibody per 1 mg of protein and 10 µL of Dynabeads Protein G (Invitrogen) overnight at 4°C. The beads were washed four times for 15 min in 500 µL of NP40 buffer and prepared for Western blotting.

### FACS analysis

For AnnexinV-PI stainings, cells were treated or transfected with siRNA as described above and harvested at the indicated time points with trypsin. Next, the samples were incubated with incubation buffer (10 mM HEPES at pH 7.4, 140 mM NaCL, 2.5 mM CaCl_2_) supplemented with Annexin-V-FLUOS (Roche, catalog no. 11828681001) and 0.1% propidium iodide solution (Sigma-Aldrich, catalog no. P4864) for 15 min at room temperature. The cells were then dissolved in incubation buffer and analyzed for active AnnexinV-PI staining by flow cytometry on a FACSCalibur (Becton Dickinson) using Cell Quest software.

For cell cycle analysis, cells were transfected with siRNA for 24 h, irradiated with 3 Gy, and harvested at the indicated time points with trypsin. The cells were washed in PBS and fixed with 4% PFA overnight at room temperature. PFA was removed by adding 95% ethanol for 1 h followed by rehydration in distilled water for 1 h. The samples were then stained with DAPI solution and analyzed by flow cytometry on a FACSCalibur (Becton Dickinson) using Cell Quest software.

### HR and NHEJ assays

Seventy-thousand U2OS (DR-GFP) and U2OS (EJ5-GFP) cells were seeded into six-well plates. Twenty-four hours later, cells were treated with the indicated siRNAs and, 8 h later, transfected with 1 μg of an I-SceI vector using Lipofectamine 2000 (Invitrogen). The next day, the medium was changed; 24 h after this, cells were harvested by trypsination and washed with PBS, and the GFP signal arising from the recombination event was measured by flow cytometry on a FACSCalibur (as described above), with fluorescence detected in the FL1-H channel (logarithmic scale). The frequency of repair in cells transfected with the various siRNAs was calculated relative to cells transfected with control siRNA. Each data point represents the average ± standard deviation from three independent experiments.

For cells overexpressing WRAP53β, 300,000 cells were seeded into six-well plates. Twenty-four hours later, cells were transfected with an I-SceI vector together with a vector expressing Flag-empty or Flag-WRAP53β using Lipofectamine 2000 (Invitrogen). The next day, the medium was changed; 24 h after this, cells were harvested by trypsination and washed with PBS, and the GFP signal arising from the recombination event was measured by flow cytometry, as above.

### Comet assay

The alkaline comet assay was performed using the method described previously with some modifications (Sasaki et al. 1997). Briefly, cells were transfected with siRNA as described above for 24 h and irradiated. After 1 h or 4 h, cells were harvested with trypsin and washed. Fifty-thousand cells were diluted in 125 μL of PBS, of which 30 µL was mixed with 70 µL of low-melting-point agarose (1% [w/v] in PBS) to a final concentration of 0.75%. The resulting suspension was laid on the top of a previously prepared normal-melting-point agarose (1% [w/v] in PBS) on frosted slides. The slides were then immersed in lysis buffer (2.5 M NaCl, 100 mM sodium EDTA, 10 mM Tris-HCl at pH 10) for 1 h at 4°C in the dark. After lysis, slides were placed in alkaline electrophoresis buffer (0.3 M NaOH, 1 mM sodium-EDTA) for 20 min at 4°C to denature DNA and express alkali-labile sites. Electrophoresis was carried out for 10 min at 4°C at 1.6 V per minute. The slides were then washed twice in neutralizing buffer (0.4 M Tris-HCl at pH 7.4) for 5 min. DNA was stained with 20 μg/mL ethidium bromide per slide. More than 100 nuclei on each slide were examined for the presence of comet tails at 200× magnification. Comet images were analyzed using CometScore software (TriTek Corporation), and the tail moment was used as the primary measurement for the quantification of DNA damage. For the neutral comet assay, the cell suspension (∼13,000 cells in 30 μL of 1× PBS) was mixed with 70 μL of 1% low-melting-point agarose type VII at a final concentration of 0.7%. The suspensions were cast on microscope slides precoated with 1% regular agarose type IA and allowed to solidify for 30 min at 4°C. After solidification, the slides were left for 2 h at 4°C in the dark in lysing buffer. The lysing buffer consisted of 2.5 M NaCl, 100 mM EDTA, 10 mM Tris–HCl, and 1% *N*-lauroylsarcosine (pH 9.5). Immediately before use, 0.5% Triton X-100 and 10% dimethylsulphoxide (DMSO) were added to the buffer and mixed for 20 min. After lysis, the slides were washed three times (30 min each wash) with cold electrophoresis buffer (1× TBE at pH 8.3), left in a fresh portion of the buffer for 1 h, and then placed in a horizontal gel electrophoresis unit filled with a fresh electrophoretic buffer. The slides were electrophoresed for 20 min at 8°C at 20 V (8 mA). After electrophoresis, the slides were rinsed with 0.4 M Tris (pH 7.5) three times for 5 min, stained with 2.5 μg/mL ethidium bromide for 20 min, and then washed three times (5 min each wash) with water and dried.

### Pulse-field gel electrophoresis

Following siRNA treatment for 24 h and irradiation, 1 × 10^6^ cells were resuspended in a solution containing 0.15 mM NaCl, 2 mM KH_2_PO_4_ (pH 6.8), 1 mM EDTA, and 5 mM MgCl_2_. An equal volume of liquefied 1% low-melting-point agarose solution in the same buffer was added to this suspension while gently mixing. The mixture was then aliquoted into gel plug-casting forms. The resulting agarose blocks were transferred into a solution containing 10 mM NaCl, 10 mM Tris-HCl (pH 9.5), 25 mM EDTA, 1% lauroylsarcosine, and 200 μg/mL proteinase K and incubated for 24 h at 50°C with continuous agitation. The plugs were rinsed three times for periods of 2 h at 4°C in 10 mM Tris-HCl (pH 8.0), and 1 mM EDTA. Subsequently, the plugs were stored until use at 4°C in 50 mM EDTA (pH 8.0). Plugs were then introduced into the 1% agarose gel, and PFGE was carried out using a horizontal gel chamber, a power supply, and a Switchback pulse controller (Hoefer Scientific Instruments). Electrophoresis was run at 12°C at 180 V in 0.5× TBE (45 mM Tris, 1.25 mM EDTA, 45 mM boric acid at pH 8.0), with the ramping rate changing from 0.8 to 30 sec over a 24-h period, applying a forward to reverse ratio of 3:1. DNA size calibration was performed using two sets of pulse markers with different size ranges, chromosomes from *Saccharomyces cerevisiae* (225–2200 kb), and a mixture of *λ*DNA HindIII fragments, *λ*DNA, and *λ*DNA concatemers (0.1–200 kb) (Sigma). DNA was stained with ethidium bromide, visualized using a 305-nm UV light source, and photographed using Polaroid 665 positive/negative film.

## Supplementary Material

Supplemental Material
