# Supplementary material for: The scaffold protein WRAP53β orchestrates the ubiquitin response critical for DNA double-strand break repair
Source: Genes Dev. 2014 Dec 15;28(24):2726–38. doi: 10.1101/gad.246546.114 (PMC4265676; doi:10.1101/gad.246546.114)
Supplement: Supplemental Material [file supp_28_24_2726__index.html]

Supplemental Material 

# The scaffold protein WRAP53β orchestrates the ubiquitin response critical for DNA double-strand break repair

## Supplemental Material

**Files in this Data Supplement:**

- Supplemental Figures.pdf
- Supplemental Table.docx
- Supplemental Text.docx
