# Supplementary figures and images for: The scaffold protein WRAP53β orchestrates the ubiquitin response critical for DNA double-strand break repair
Source: Genes Dev. 2014 Dec 15;28(24):2726–38. doi: 10.1101/gad.246546.114 (PMC4265676; doi:10.1101/gad.246546.114)

**A**

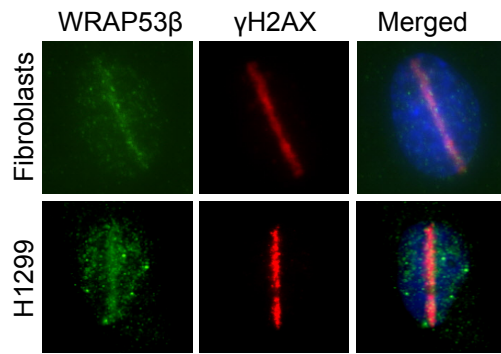

**B**

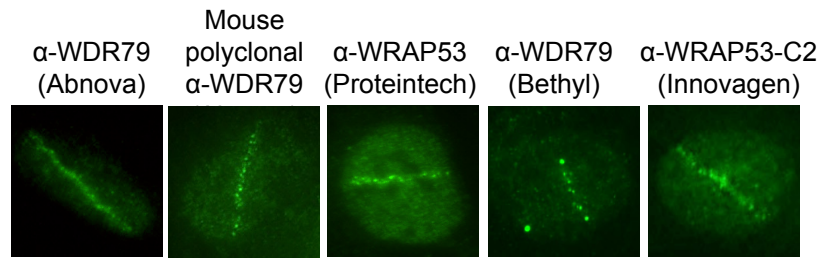

**C**

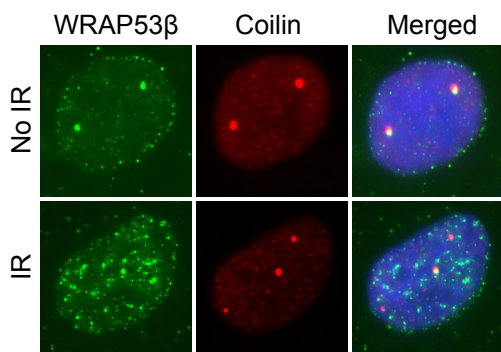

**D**

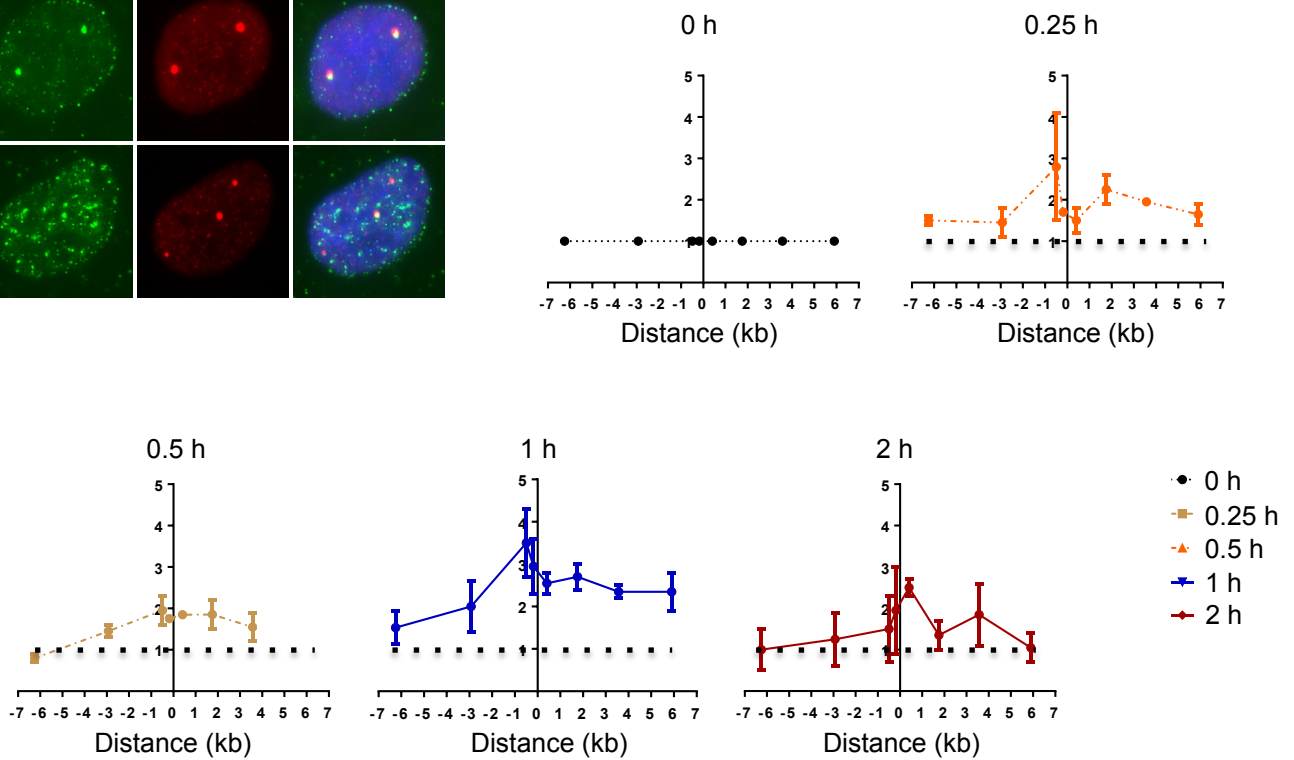

**A**

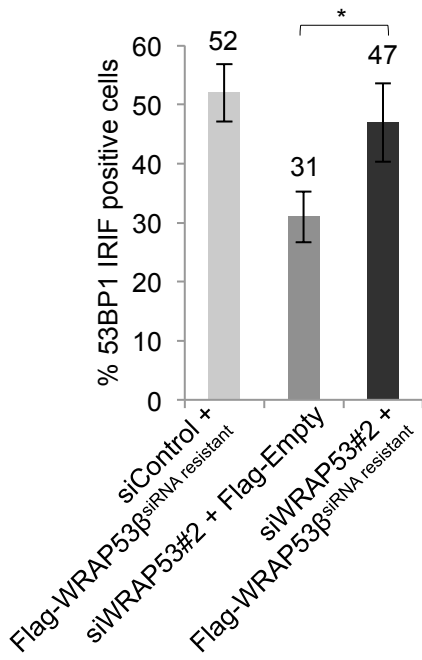

**B**

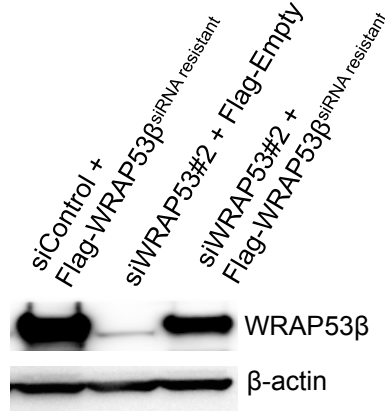

**C**

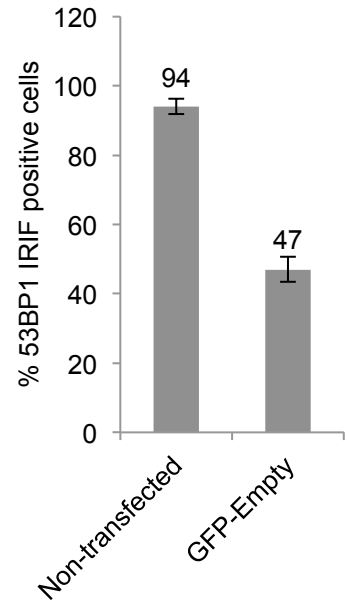

**D**

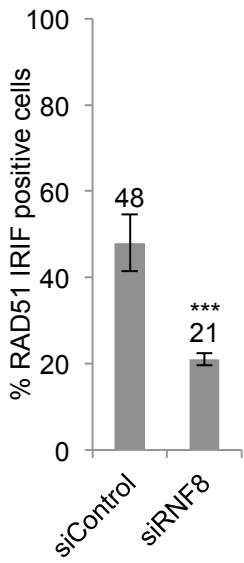

**E**

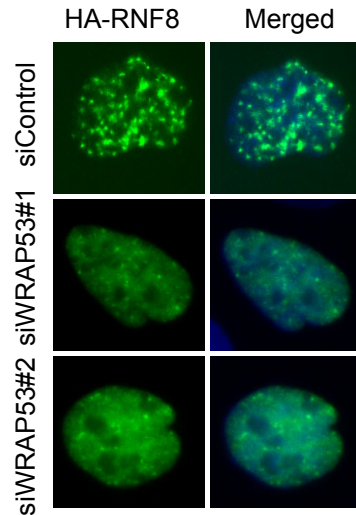

**F**

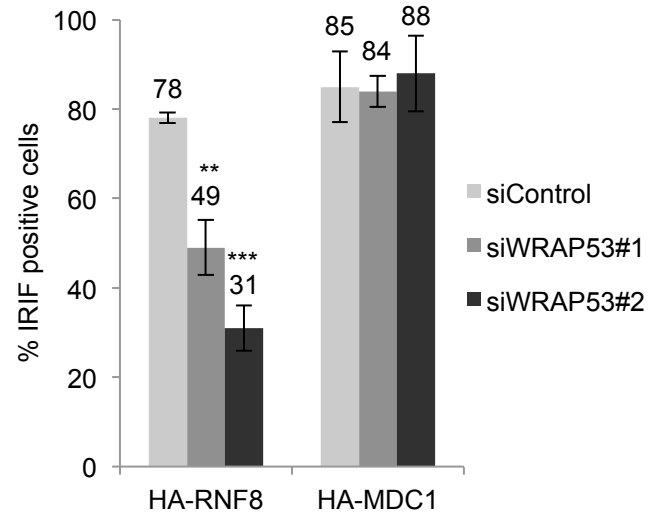

**G**

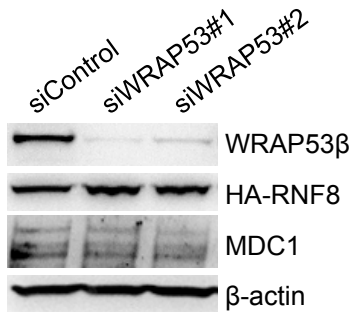

**H**

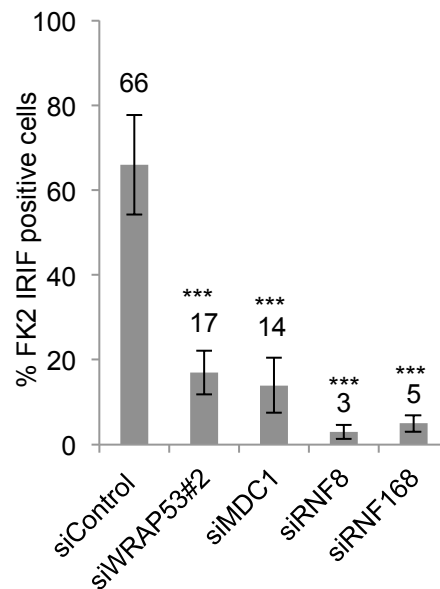

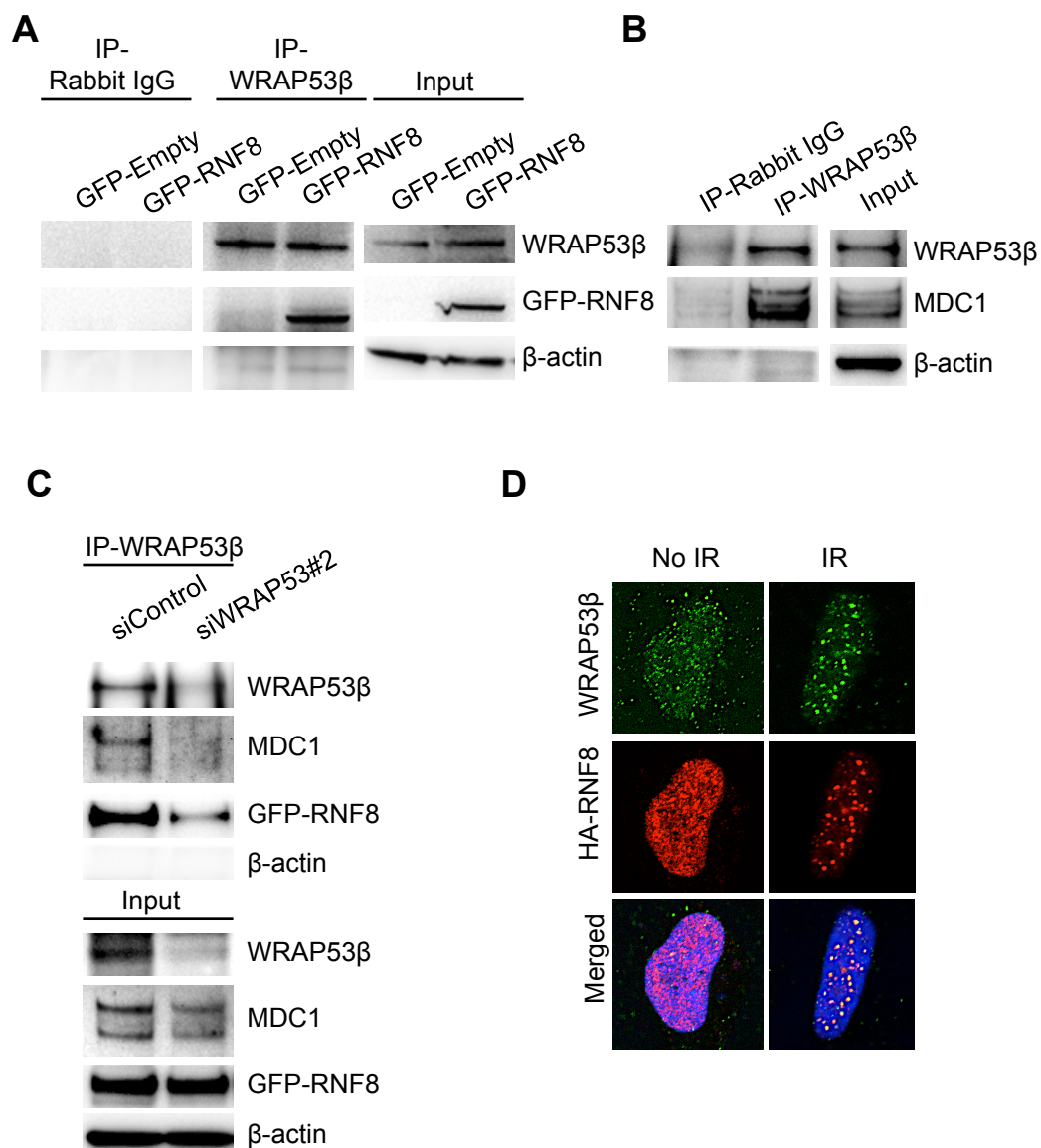

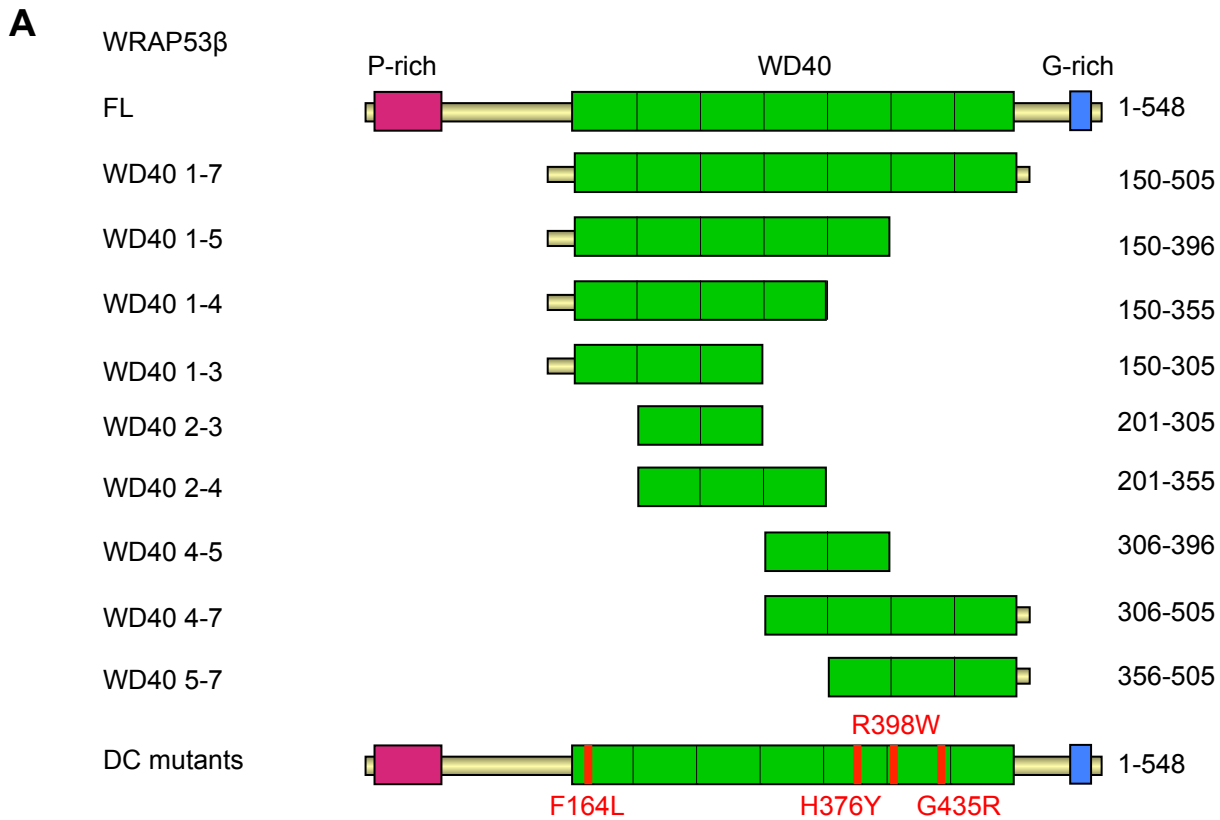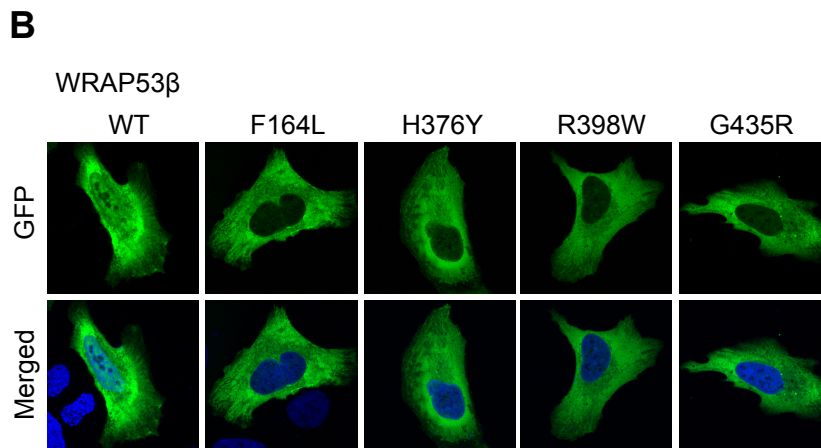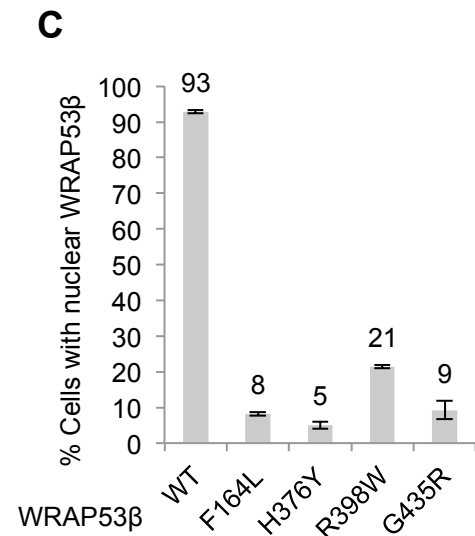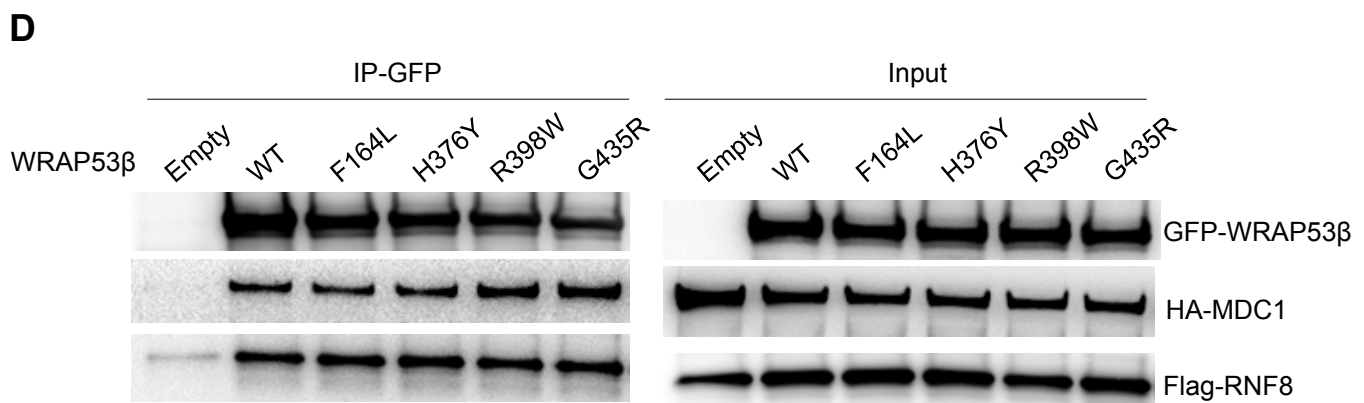

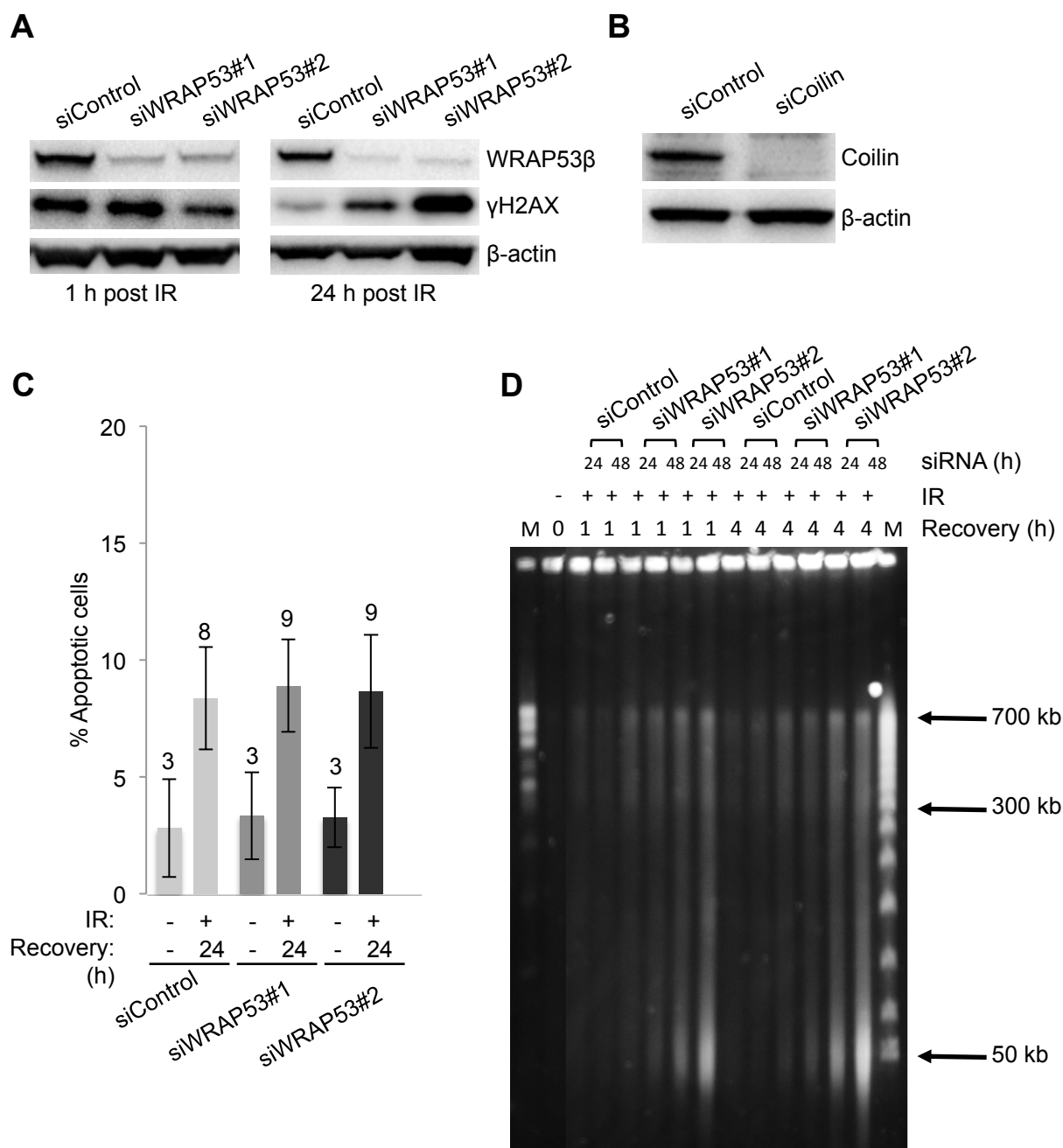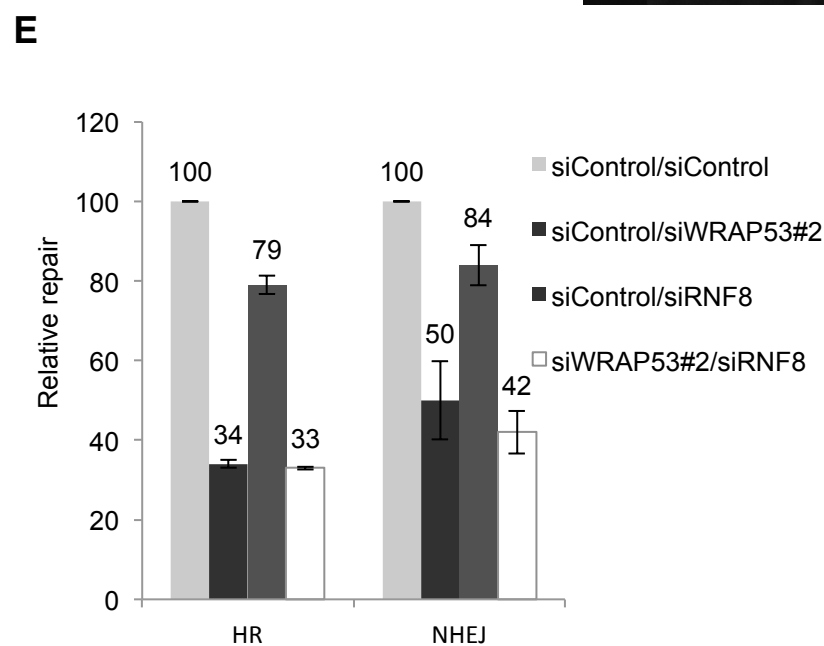

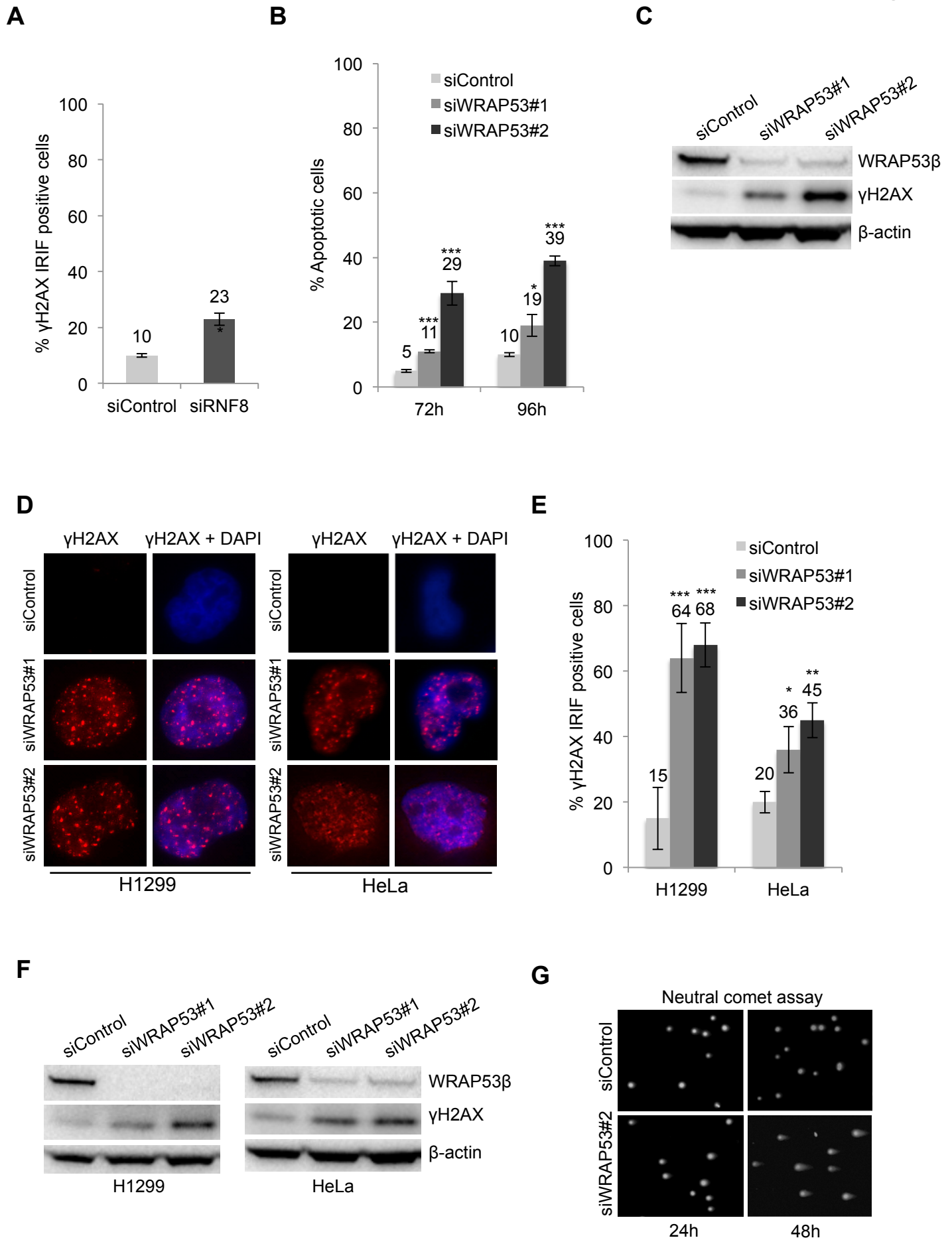

Supplement: Supplemental Material [file supp_28.24.2726_Supplemental_Figures.pdf]
