# Supplementary material for: The scaffold protein WRAP53β orchestrates the ubiquitin response critical for DNA double-strand break repair
Source: Genes Dev. 2014 Dec 15;28(24):2726–38. doi: 10.1101/gad.246546.114 (PMC4265676; doi:10.1101/gad.246546.114)
Supplement: Supplemental Material [file supp_28.24.2726_Supplemental_Table.docx]

**Supplementary Table 1** Primers used in this study

| PCR primer name | Sequence 5’- 3’ |
| --- | --- |
| Mutagenesis primers for WRAP53β | |
| Flag-WRAP53β^siRNA resistant^ F | GCAAACGGGAGTCTCTCTGAAGAAGAAGC |
| Flag-WRAP53β^siRNA resistant^ R | GCTTCTTCTTCAGAGAGACTCCCGTTTGC |
| EGFP-WRAP53β F164L F | GGTTCCTGGTCAGAGTTAAGCACCCAACCTGA |
| EGFP-WRAP53β F164L R | TCAGGTTGGGTGCTTAACTCTGACCAGGAACC |
| EGFP-WRAP53β H376Y F | GCATCACCCACCTCTGCTTTTATCCCGATGGC |
| EGFP-WRAP53β H376Y R | GCCATCGGGATAAAAGCAGAGGTGGGTGATGC |
| EGFP-WRAP53β R398W F | CTGTGCTGGGATCTCTGGCAGTCTGGTTACC |
| EGFP-WRAP53β R398W R | GGTAACCAGACTGCCAGAGATCCCAGCACAG |
| EGFP-WRAP53β G435R F | GGCAGCACGAGCAGGGCTGTCTCTG |
| EGFP-WRAP53β G435R R | CAGAGACAGCCCTGCTCGTGCTGCC |
| Cloning primers for WRAP53β | |
| EGFP-WRAP53β FL1 F | GAATTCT ATGAAGACTTTGGAGACTCAACCGTTA |
| EGFP-WRAP53β FL548 R | GGTACC TTATATCAGCTCACCCACACCTCC |
| EGFP-WRAP53β N160 R | GGTACCTTA GGAACCACTGAGAAATCGAGGC |
| EGFP-WRAP53β WD40-1 150 F | CTCGAGGAATTCT TTCTCCCAGCTGCCTCGATTTC |
| EGFP-WRAP53β WD40-2 201 F | CTCGAGGAATTCT GAGCTGTACCATGAGGGGGAGC |
| EGFP-WRAP53βWD40-4 306 F | CTCGAGGAATTCT GCCCGGCCTGGCCGAGACTGCG |
| EGFP-WRAP53β WD40-5 356 F | CTCGAGGAATTCT GATGGCTCCCCTCTCGCCTTGC |
| EGFP-WRAP53β WD40-3 305 R | GGGAAGCTTGGTACCTTA CGTGGAAAAAACACGCACAGTCC |
| EGFP-WRAP53β WD40-4 355 R | GGGAAGCTTGGTACCTTA ATCCCAGGCATACAGACCCAG |
| EGFP-WRAP53β WD40-5 396 R | GGGAAGCTTGGTACCTTA ATCCCAGCACAGGAGCTCAGCA |
| EGFP-WRAP53β WD40-7 505 R | GGGAAGCTTGGTACCTTA CGTGGAGAGCAAGGGAAGGCCC |
| EGFP-WRAP53β C500 F | GAATTCT CTTCCCTTGCTCTCCACGCG |
| Cloning primers for RNF8 | |
| EGFP-RNF8 FL1 F | CTCGAGGAATTCT ATGGGGGAGCCCGGCTTCTTC |
| EGFP-RNF8 FL485 R | GGGGGGTACC TCAGAACAATCTCTTTGCTTT |
| EGFP-RNF8 N111 R | AAGCTTGGTACCTTA AGGCACTCCAAGTTGGATGTA |
| EGFP-RNF8 M112 F | CTCGAGGAATTCT CTGGAAAATAAGGAGAATGCG |
| EGFP-RNF8 M401 R | GGGGGGTACCTTA GAGCTCATTCTCTAGCACATC |
| EGFP-RNF8 C402 F | CTCGAGGAATTCT CAATGTATTATTTGTTCAGAA |
| 3xFlag-RNF8 FL1 F | GGGGGAATTCA ATGGGGGAGCCCGGCTTCTTC |
| 3xFlag-RNF8 FL485 R | GGGGGGTACC TCAGAACAATCTCTTTGCTTT |
| 3xFlag-RNF8 N111 R | AAGCTTGGTACCTTA AGGCACTCCAAGTTGGATGTA |
| 3xFlag-RNF8 N140 R | GGGGGGTACCTTA ATTCTTTGGGGAAAGACAAGG |
