# Supplementary material for: The scaffold protein WRAP53β orchestrates the ubiquitin response critical for DNA double-strand break repair
Source: Genes Dev. 2014 Dec 15;28(24):2726–38. doi: 10.1101/gad.246546.114 (PMC4265676; doi:10.1101/gad.246546.114)
Supplement: Supplemental Material [file supp_28.24.2726_Supplemental_Text.docx]

**SUPPLEMENTAL MATERIAL**

**The WD40 protein WRAP53β regulates RNF8-mediated repair of DNA double-strand breaks** Sofia Henriksson^1^, Hanif Rassoolzadeh^1,5^, Elisabeth Hedström^1,5^, Christos Coucoravas^1^, Alexander Julner^1^, Michael Goldstein^2^, Gabriela Imreh^3^, Boris Zhivotovsky^3^, Michael B. Kastan^2^, Thomas Helleday^4^, and Marianne Farnebo^1^

This supplemental material contains:

Supplementary Figures 1-6

Supplementary Table 1

**Supplementary Figure 1** WRAP53β accumulates at DSBs. (A) Human fibroblasts and H1299 cells were micro-irradiated, fixed 5 min later and immunostained for WRAP53β and γH2AX. (B) U2OS cells were micro-irradiated, fixed 5 min later and immunostained for WRAP53β using the indicated antibodies. (C) U2OS cells were either left untreated or irradiated (6 Gy); and 1 h later fixed after pre-extraction with CSK buffer and immunostained for WRAP53β and coilin. (D) ChIP assay monitoring the kinetics of WRAP53β recruitment to the I-PpoI-induced DSB at chromosome 1 in MCF7 cells stably expressing ddI-PpoI. Time indicated is h after the addition of 4-OHT. The I-PpoI cleavage site on chromosome 1 is located at distance 0. Data are shown as mean of two independent experiments. The y-axis displays the fold change in relative occupancy normalized to the control.

**Supplementary Figure 2** WRAP53β knockdown impairs recruitment of repair proteins to DSBs and introduction of siRNA resistant WRAP53β restores 53BP1 foci. (A) U2OS cells were transfected with siControl or siWRAP53#2 oligonucleotides (for 8 h); followed by transfection of Flag-Empty or Flag-WRAP53β^siRNA resistant^ plasmids (for 16 h), exposed to IR (6 Gy); and after 1 h immunostained for 53BP1 and Flag followed by quantification of the results or (B) harvested for western blotting using the indicated antibodies. The graph in (A) shows the percentage of 200 Flag-transfected cells in each experiment whose nuclei were 53BP1-positive. (C) Percentage of 53BP1 foci positive cells in non-transfected and GFP-empty vector positive cells immunostained 1 h after exposure to 6 Gy of IR, showing that plasmid transfection per se results in a lower number of 53BP1 foci positive cells compared to non-transfected cells. (D) U2OS cells were treated with the siRNAs indicated for 24 h; followed by irradiation (6 Gy); allowed to recover for 1 h; fixed and immunostained for RAD51. (E) U2OS cells were treated with the siRNAs indicated (for 48 h) and HA-RNF8 (for 6 h); followed by irradiation (6 Gy); allowed to recover for 1 h; and immunostained for HA-RNF8 (F) quantification of the cells with HA-RNF8 IRIF treated as (E) and of cells with HA-MDC1 IRIF treated with the siRNAs indicated. The results show the percentage of cells whose nuclei contained IRIF (n=100). (G) Cells treated as (F) were also harvested for western blotting of WRAP53β, HA-RNF8, MDC1 and β-actin. (H) U2OS cells were treated with the siRNAs indicated for 48 h followed by irradiation (6 Gy); allowed to recover for 1 h; and immunostained for FK2; followed by quantification. The percentage of nuclei containing >10 FK2 foci was quantified in the 100 cells counted for each experiment (n=3). The error bars depict the s.e.m.; n=3, * p<0.05, ** p<0.01, *** p<0.001, as determined by Student’s t-test.

**Supplementary Figure 3** WRAP53β binds GFP-RNF8 and MDC1 (A) U2OS cells were transiently transfected with GFP-Empty or GFP-RNF8 plasmids for 16 h; irradiated (6 Gy); and allowed to recover for 30 min. Total input, IgG- and WRAP53β-immunoprecipitates were analyzed by western blotting of WRAP53β, GFP-RNF8 and β-actin. (B) IP using rabbit IgG or WRAP53β antibodies in irradiated (6 Gy, 30 min recovery) U2OS cells followed by immunoblotting with the indicated antibodies. (C) IP using WRAP53β antibody in irradiated (6 Gy, 15 min recovery) U2OS cells treated with the indicated siRNA (for 48 h) and transfected with GFP-RNF8 (for 16 h); followed by immunoblotting with the indicated antibodies. (D) U2OS cells were transiently transfected with HA-RNF8 plasmids for 6 h, left untreated or irradiated (6 Gy); allowed to recover for 1 h; fixed after pre-extraction with CSK buffer and immunostained for WRAP53β and HA-RNF8.

**Supplementary Figure 4** Dyskeratosis congenita-associated WRAP53β mutations results in nuclear exclusion of WRAP53β. (A) Schematic illustration of EGFP-tagged constructs of WRAP53β. (B) U2OS cells were transiently transfected with the EGFP-plasmids indicated for 16 h; fixed and assessed for GFP signal. (C) Quantification of the results in (B), showing the percentage of nuclei with GFP-WRAP53β (n=200). (D) U2OS cells were transiently transfected with the EGFP-plasmids indicated, Flag-RNF8 and HA-MDC1 for 16 h; irradiated; subjected to IP of GFP; followed by immunoblotting for HA-MDC1, Flag-RNF8 and GFP-WRAP53β.

**Supplementary Figure 5** WRAP53β depletion induces persistent H2AX phosphorylation without affecting cell death and leads to accumulation of IR-induced DSBs. (A) U2OS cells were treated with siControl, siWRAP53#1 and siWRAP53#2 oligonucleotides for 24 h; irradiated (6 Gy); and analyzed by western blotting for WRAP53β, γH2AX and β-actin expression at the indicated time points. (B) U2OS cells were treated with siControl and siCoilin oligonucleotides for 48 h and analyzed by western blotting for coilin and β-actin expression. (C) AnnexinV-PI FACS analysis of U2OS cells treated with the indicated siRNAs for 24 h, left untreated or irradiated (6 Gy) for an additional 24 h. (D) WRAP53β depletion leads to increased accumulation of DSBs after irradiation. Formation of DNA fragments assessed by PFGE in cells treated with siControl, siWRAP53#1 and siWRAP53#2 oligonucleotides for 24 h or 48 h; exposed to irradiation (10 Gy); followed by 1 h or 4 h recovery. (E) HR and NHEJ efficiency, respectively, following treatment of the cells with the siRNA indicated for 48 h. The error bars depict the s.e.m.; n=3.

**Supplementary Figure 6** WRAP53β depletion induces spontaneous H2AX phosphorylation and accumulation of DSBs. (A) U2OS cells were treated with the siRNAs indicated for 24 h; fixed and then immunostained for γH2AX; followed by quantification. The percentage of nuclei containing >10 γH2AX foci was quantified in the 100 cells counted for each experiment (n=3). (B) AnnexinV-PI FACS analysis of U2OS cells treated with the indicated siRNAs for 72 h or 96 h. (C) U2OS cells were treated with the siRNAs indicated for 72 h; and analyzed by western blotting for WRAP53β, γH2AX and β-actin expression. (D) H1299 and HeLa cells were treated with the siRNAs indicated for 24 h; fixed and then immunostained for γH2AX. (E) Quantification of the results in (D), as the percentage of 200 cells counted in each experiment whose nuclei contained >10 γH2AX foci. (F) H1299 and HeLa cells were treated with the siRNAs indicated for 24 h; and harvested for western blot analysis of WRAP53β, γH2AX and β-actin. (G) DNA damage was assessed by the neutral comet assay in U2OS cells treated with siWRAP53#2 or siControl for 24 or 48 h. Error bars depict the s.e.m.; n=3, * p<0.05, ** p<0.01, *** p<0.001, Student’s t-test.
